# A gain-of-function p53 mutant synergizes with oncogenic NRAS to promote acute myeloid leukemia in mice

**DOI:** 10.1172/JCI173116

**Published:** 2023-12-15

**Authors:** Adhithi Rajagopalan, Yubin Feng, Meher B. Gayatri, Erik A. Ranheim, Taylor Klungness, Daniel R. Matson, Moon Hee Lee, Mabel Minji Jung, Yun Zhou, Xin Gao, Kalyan V.G. Nadiminti, David T. Yang, Vu L. Tran, Eric Padron, Shigeki Miyamoto, Emery H. Bresnick, Jing Zhang

**Affiliations:** 1 McArdle Laboratory for Cancer Research, University of Wisconsin–Madison, Madison, Wisconsin, USA.; 2 Department of Pathology and Laboratory Medicine, University of Wisconsin School of Medicine and Public Health, Madison, Wisconsin, USA.; 3 University of Wisconsin Carbone Cancer Center, Madison, Wisconsin, USA.; 4 Wisconsin Blood Cancer Research Institute, Department of Cell and Regenerative Biology, University of Wisconsin School of Medicine and Public Health, Madison, Wisconsin, USA.; 5 Division of Hematology, Medical Oncology and Palliative Care, Department of Medicine, University of Wisconsin, Madison, Wisconsin, USA.; 6 Chemical Biology and Molecular Medicine Program, Moffitt Cancer Center, Tampa, Florida, USA.

**Keywords:** Hematology, Leukemias

## Abstract

We previously demonstrated that a subset of acute myeloid leukemia (AML) patients with concurrent RAS pathway and *TP53* mutations have an extremely poor prognosis and that most of these *TP53* mutations are missense mutations. Here, we report that, in contrast to the mixed AML and T cell malignancy that developed in *Nras^G12D/+^* *p53^–/–^* (NP^–/–^) mice, *Nras^G12D/+^* *p53^R172H/+^* (NP^mut^) mice rapidly developed inflammation-associated AML. Under the inflammatory conditions, NP^mut^ hematopoietic stem and progenitor cells (HSPCs) displayed imbalanced myelopoiesis and lymphopoiesis and mostly normal cell proliferation despite MEK/ERK hyperactivation. RNA-Seq analysis revealed that oncogenic NRAS signaling and mutant p53 synergized to establish an NP^mut^-AML transcriptome distinct from that of NP^–/–^ cells. The NP^mut^-AML transcriptome showed GATA2 downregulation and elevated the expression of inflammatory genes, including those linked to NF-κB signaling. NF-κB was also upregulated in human *NRAS* *TP53* AML. Exogenous expression of GATA2 in human NP^mut^ KY821 AML cells downregulated inflammatory gene expression. Mouse and human NP^mut^ AML cells were sensitive to MEK and NF-κB inhibition in vitro. The proteasome inhibitor bortezomib stabilized the NF-κB–inhibitory protein IκBα, reduced inflammatory gene expression, and potentiated the survival benefit of a MEK inhibitor in NP^mut^ mice. Our study demonstrates that a p53 structural mutant synergized with oncogenic NRAS to promote AML through mechanisms distinct from *p53* loss.

## Graphical abstract

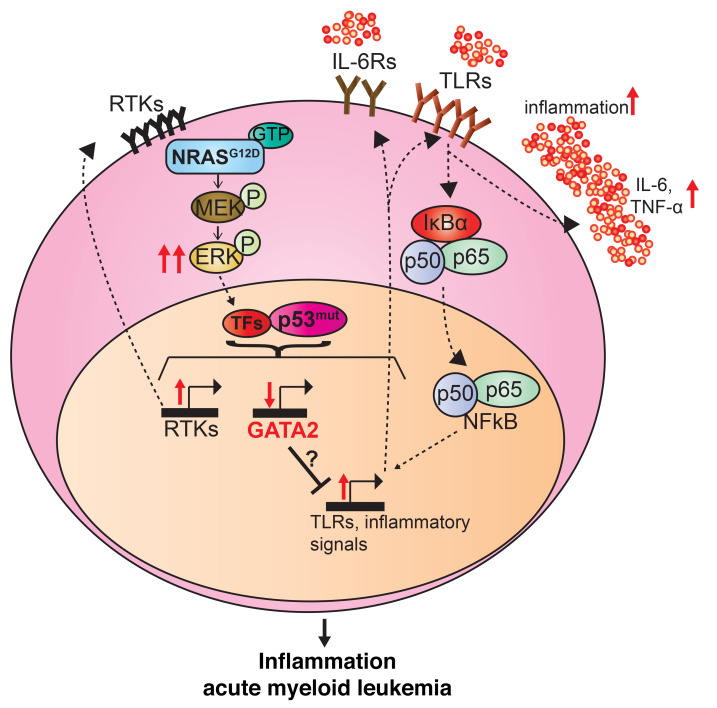

## Introduction

Acute myeloid leukemia (AML) is an aggressive and devastating hematologic malignancy characterized by the accumulation of partially differentiated myeloid blast cells (≥20%) in bone marrow (BM) and/or other hematopoietic organs, leading to BM failure and death (1). Patients with AML can be broadly grouped into 3 distinct categories: (a) secondary AML (s-AML), which results from acute transformation of chronic myeloid disease, such as myelodysplastic syndrome (MDS), myeloproliferative neoplasm (MPN), or mixed MPN/MDS (e.g. chronic myelomonocytic leukemia [CMML]); (b) therapy-related AML (t-AML), which occurs in patients who were previously treated with chemo/radiation therapies; and (c) de novo AML, which is not preceded by a known hematologic disorder or therapy exposure (2). In general, patients with s-AML or t-AML have inferior survival rates relative to patients with de novo AML (3, 4). The adverse outcome in t-AML is driven predominantly by the increased frequency of *TP53* mutations, as patients with t-AML who do not have*TP53* mutations have a median survival that approximates that of patients with de novo AML.

Hyperactivating RAS pathway mutations, including oncogenic *NRAS* and *KRAS*, are prevalent in all 3 categories of AML but differ in the disease stages at which they arise. In de novo AML, *NRAS* and *KRAS* mutations are usually acquired later in clonal evolution to drive AML progression (5). By contrast, they are commonly found as early mutations in clonal hematopoiesis in patients after chemo/radiation therapies (6) and are prevalent in t-AML (7). Thus, they may serve as initiation or progression mutations in t-AML. Recently, we and others found that *NRAS* mutations associate with and potentially promote the transformation of MDS and CMML to s-AML (2, 8–10).

*TP53* encodes a master transcription factor that regulates cell proliferation and apoptosis in response to DNA damage and other cellular stresses (11). *TP53* mutations are most closely associated with de novo AMLs harboring complex karyotypes and t-AMLs, but can also be seen in s-AMLs. In all groups of AML they are linked to poor prognosis (12, 13). This adverse risk is compounded by co-mutation of RAS pathway genes (*NRAS*, *KRAS*, *BRAF*, *NF1*, *PTPN11*, and/or *CBL*), leading to a dismal overall AML survival of less than 5 months (9, 14–16). Most of these patients with double mutations harbored heterozygous *TP53* missense mutations. These data suggest that *TP53* and RAS pathway mutations may cooperate to promote AML in humans.

Cancer-associated *TP53* mutations include 2 major classes: loss of *TP53* via genetic deletion of the *TP53* locus and missense mutations predominantly occurring in the DNA-binding domain (11). In solid tumors, p53 mutants, including the hotspot structural mutant R175H, not only attenuate the capacity to activate WT p53 target genes but also display neomorphic gain-of-function (GOF) activities to promote tumorigenesis beyond p53 loss (17). In the hematopoietic system, the consequences of mutant p53 remain less clear. Boettcher et al. proposed a dominant-negative effect for missense p53 mutants in myeloid malignancies (18). By contrast, the p53 R248W mutant promotes hematopoietic stem cell (HSC) self-renewal through its GOF interaction with EZH2 (19), whereas the p53 R172H mutant (corresponding to human R175H) exhibits GOF activity in AML via activation of the embryonic transcription factor (TF) Foxh1 (20). In the human KY821 AML cell line carrying concurrent oncogenic *NRAS* and *TP53^R175H/–^* mutations, sustained expression of mutant p53 is required to maintain AML cells in vitro and in vivo (20).

We previously tested genetic interactions between *Nras^G12D^* and *p53^–/–^* (14) and found that *Nras^G12D/+^* *p53^–/–^* (referred to hereafter as NP^–/–^) mice developed a mixed AML and T cell lymphoma/leukemia. The NP^–/–^ AML transcriptome is predominantly regulated by p53 loss. In this study, we investigate genetic interactions between oncogenic NRAS signaling and the p53 R172H mutant. Our data demonstrate that *Nras^G12D/+^* *p53^mut/+^* (NP^mut^) rapidly induced AML characterized by inflammation and cellular and molecular mechanisms distinct from those of NP^–/–^.

## Results

### Mutant p53 cooperates with oncogenic NRAS to rapidly induce AML.

**Figure 1:**
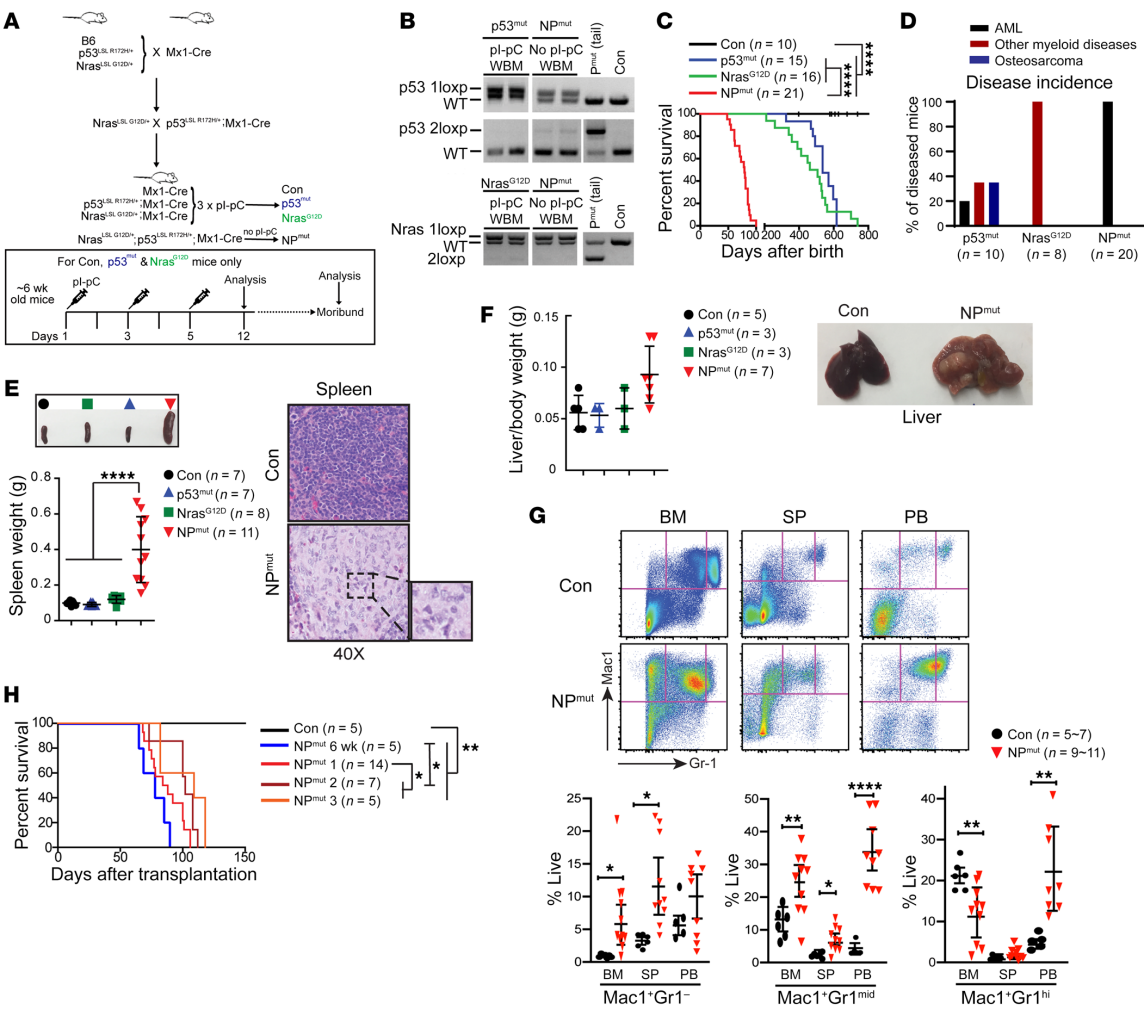
NP^mut^ mice rapidly develop lethal AML. (**A**) Transgenic mouse lines and illustration of the Mx1-Cre induction scheme. (**B**) Genotyping of *p53* and *Nras* alleles in non–pl-pC–injected NP^mut^ and pI-pC–injected p53^mut^ (P^mut^) and Nras^G12D^ BM cells. WBM, whole bone marrow. (**C**) Kaplan-Meier survival curves of all 4 groups of mice. (**D**) Disease incidence in moribund p53^mut^, Nras^G12D^, and NP^mut^ mice. (**E**) Quantification of SP weight and H&E-stained SP sections to show monocytic leukemia cells. Original magnification, ×1 (top panel), ×40 (inset). (**F**) Quantification of liver/body weight and representative image of gross liver morphology. (**G**) Quantification of monocytes (Mac1^+^Gr1^–^), myeloid precursors (Mac1^+^Gr1^mid^), and neutrophils (Mac1^+^Gr1^hi^) in BM, SP, and PB. (**E**–**G**) Results are presented as the mean ± SD. (**H**) Kaplan-Meier survival curves of recipient mice transplanted with BM cells from 6-week-old NP^mut^ mice and with NP^mut^ AML cells from 3 representative donors. *P < 0.05, **P < 0.01, and ****P < 0.0001, by log-rank test followed by Benjamini-Hochberg multiple-comparison analysis (**C** and **H**), 1-way ANOVA followed by Tukey’s post hoc test (**E** and **F**), and unpaired, 2-tailed Student’s *t* test (**G**). Con, control.

To explore potential genetic interactions between p53 missense mutant and oncogenic NRAS signaling, we used *Vav-Cre* to activate both mutations since E11.5 (21, 22). The compound mice rapidly died from AML within a few weeks of birth (Supplemental Figure 1A; supplemental material available online with this article; https://doi.org/10.1172/JCI173116DS1). We then switched to the inducible *Mx1-Cre* line and generated *Mx1-Cre* (control), *p53^LSL-R172H/+^* *Mx1-Cre* (p53^mut^), *Nras^LSL-G12D/+^* *Mx1-Cre* (Nras^G12D^), and *Nras^LSL-G12D/+^* *p53^LSL-R172H/+^* *Mx1-Cre* (NP^mut^) mice. Six-week-old mice were administered polyinosinic-polycytidylic acid (pI-pC) to induce the expression of oncogenic *Nras* and mutant *p53* from their respective endogenous loci. Unexpectedly, NP^mut^ mice were either found dead or became moribund within a few days after the initial pI-pC injection (Supplemental Figure 1B). This finding was reminiscent of *Kras^LSL-G12D/fl^* *Mx1-Cre* and *Kras^LSL-G12D/+^* *Dnmt3^fl/fl^* *Mx1-Cre* mice that we previously described (23, 24) and was likely due to leaky expression of Cre and amplified IFN signaling. As single-mutant mice do not have sufficient recombination efficiency without pI-pC injection (25), approximately 6-week-old control and single-mutant mice were treated with pI-pC three times every other day as previously described (26), whereas pI-pC treatment was withheld from NP^mut^ mice (Figure 1A). We found that the BM cells from moribund NP^mut^ mice expressed the recombined 1loxp *Nras^G12D^* and *p53^R172H^* alleles like the BM cells from age-matched single-mutant mice and retained the WT *Nras* and *p53* alleles (Figure 1B).

After a prolonged latency, p53^mut^ mice developed various myeloid diseases (including AML and myeloproliferative neoplasm) or osteosarcoma (median survival: ~530 days), whereas 100% of Nras^G12D^ mice developed myeloid disorders as described previously (26, 27) (median survival: ~480 days) (Figure 1, C and D). In sharp contrast to NP^–/–^ mice that developed mixed AML and T cell malignancy (14), NP^mut^ mice rapidly developed lethal AML with full penetrance (median survival, ~80 days) (Figure 1, C and D), characterized by splenomegaly and accumulation of partially differentiated myeloid blast cells in the spleen (SP) and liver (Figure 1, E and F). Flow analysis of hematopoietic tissues from moribund NP^mut^ mice indicated expansion of Mac1^+^Gr1^–^ monocytes in BM and the SP, expansion of Mac1^+^Gr1^hi^ neutrophils in peripheral blood (PB), and a reduction of neutrophils in BM (Figure 1G). By contrast, tissues from age-matched Nras^G12D^ and p53^mut^ mice did not show significant phenotypes (Figure 1, E and F, and Supplemental Figure 2). Unlike the myeloid blasts that we characterized in previous AML models (10, 14), AML blasts in NP^mut^ BM, SP, and PB corresponded to Mac1^+^Gr1^mid^ immature myeloid precursors (Figure 1G). Blood smear preparations revealed circulating atypical, immature monocytoid cells in the NP^mut^ PB (Supplemental Figure 3).

To determine whether oncogenic NRAS and mutant p53 induce AML in a cell-autonomous manner, we transplanted total BM cells from 6-week-old NP^mut^ mice along with the same number of competitor cells into irradiated recipient mice. Without pI-pC injections, the recipient mice died from AML with a latency comparable to that of primary NP^mut^ mice (Figure 1H). Moreover, NP^mut^ AML phenotypes were transplantable into irradiated recipients (Figure 1H). Taken together, our data demonstrate that mutant p53 cooperated with oncogenic NRAS to promote AML.

### NP^mut^ hematopoietic stem and progenitor cells show imbalanced myelopoiesis and lymphopoiesis.

**Figure 2:**
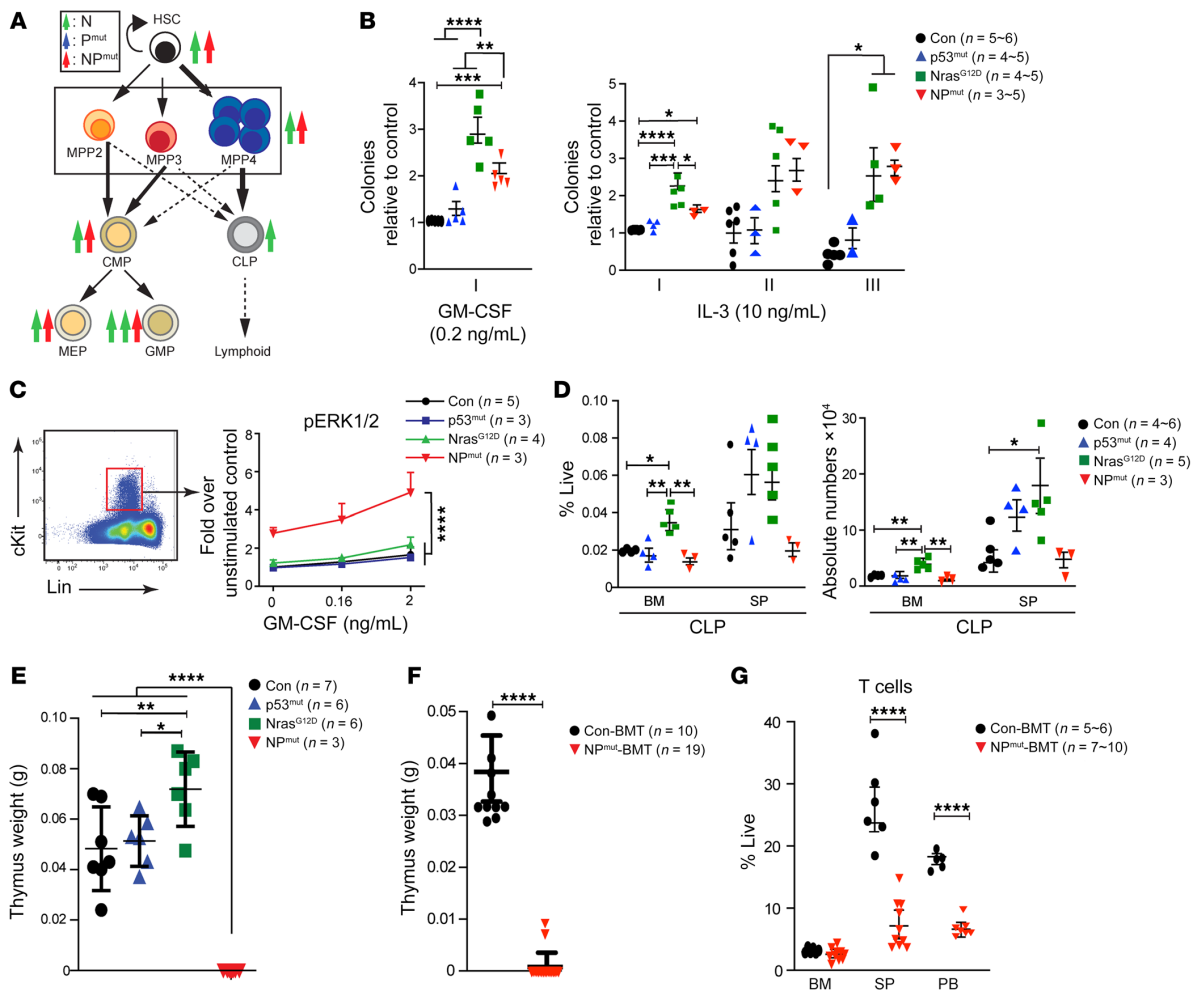
NP^mut^ HSPCs show hyperactivation of ERK signaling and reduced lymphopoiesis. (**A**–**D**) Analyses were performed in control, p53^mut^, and Nras^G12D^ mice 1 week after the last pI-pC injection and in age-matched NP^mut^ mice. (**A**) Schematic illustration of HSPC compartments, including HSCs (defined as Lin^–^Sca1^+^cKit^+^Flk2^–^CD48^–^CD150^+^), MPP2–4 (defined as described in the legend to Supplemental Figure 4), CMPs, CLPs, MEPs, and GMPs (defined as described in the legend to Supplemental Figure 5). The number of arrows indicates the overall degree of expansion or reduction versus control cells. (**B**) Quantification of myeloid colonies formed from the same number of BM cells in the presence of GM-CSF and the replating capability of BM cells in the presence of IL-3. (**C**) Quantification of p-ERK1/2 levels in Lin^–^cKit^+^ HSPCs. (**D**) Quantification of CLPs from the BM and SP. (**E**–**G**) Analyses were performed in moribund NP^mut^ mice and age-matched control mice. (**E** and **F**) Quantification of thymus weight in primary mice (**E**) and NP^mut^ recipients (**F**). (**G**) Quantification of T cells in hematopoietic tissues, including BM, SP, and PB, from NP^mut^ recipients. (**B**–**G**) Results are presented as the mean ± SD. *P < 0.05, **P < 0.01, ***P < 0.001, and ****P < 0.0001, by 1-way ANOVA followed by Tukey’s post hoc test (**B**, **D**, and **E**), 2-way ANOVA with Tukey’s post hoc test (**C**), and unpaired, 2-tailed Student’s *t* test (**F** and **G**).

To understand the cellular mechanisms underlying NP^mut^ AML, we analyzed hematopoietic stem and progenitor cells (HSPCs) from control, p53^mut^, and Nras^G12D^ mice 1 week after the last pI-pC injection and from age-matched NP^mut^ mice. Long-term HSCs (LT-HSCs), short-term HSCs (ST-HSCs), and multipotent progenitors (MPPs) 2–4 were delineated as previously described (28) (Supplemental Figure 4A). The numbers and cell-cycle profiles of LT-HSC, ST-HSC, MPP2-4, and Lin^–^Sca1^+^cKit^+^ (LSK) cells in p53^mut^ mice were indistinguishable from those in control mice (Figure 2A and Supplemental Figure 4). By contrast, the numbers of LT-HSCs and ST-HSCs were increased in Nras^G12D^ and NP^mut^ SP, while the numbers of MPP2-4 and LSK cells were elevated in Nras^G12D^ SP and NP^mut^ BM compared with those in controls (Supplemental Figure 4). However, we observed no significant differences between Nras^G12D^ and NP^mut^ HSPCs (Figure 2A and Supplemental Figure 4). As reported before (25, 29), Nras^G12D^ BM HSCs (defined as LSK CD48^–^CD150^+^) were hyperproliferative. Surprisingly, the cell-cycle profiles of NP^mut^ HSPCs were comparable to those of control cells (Supplemental Figure 4).

To investigate how p53^mut^ affects downstream progenitors in Nras^G12D^ mice, we first analyzed myeloid progenitors (MPs) and subpopulations (common myeloid progenitors [CMPs], granulocyte-macrophage progenitors [GMPs], and megakaryocyte-erythroid progenitors [MEPs]) in control, p53^mut^, Nras^G12D^, and NP^mut^ mice (Supplemental Figure 5). The MP compartment in p53^mut^ mice was comparable to that in control mice. In agreement with our previous results (25, 30), all MP compartments of Nras^G12D^ mice including CMP, GMP, and MEP compartments were significantly expanded in BM and/or SP compared with controls. Surprisingly, expression of p53^mut^ did not further expand the number of MPs in Nras^G12D^ mice. Rather, the numbers of GMPs in NP^mut^ BM and SP were lower than those in Nras^G12D^ BM and SP, leading to an overall reduced MP compartment compared with Nras^G12D^ BM and SP (Figure 2A). We further evaluated the clonogenicity of BM MPs in vitro. Nras^G12D^ and NP^mut^ cells showed enhanced colony-forming and replating capabilities, while p53^mut^ cells were similar to controls (Figure 2B). Consistent with GMP flow analyses, NP^mut^ cells formed fewer colonies than did Nras^G12D^ cells in the presence of GM-CSF or IL-3 (first round of replating). Surprisingly, in contrast to these modest NP^mut^ HSPC phenotypes, phosphorylated ERK (p-ERK) levels in NP^mut^ HSPCs were 2-fold higher than those in control, p53^mut^, and Nras^G12D^ cells in the absence or presence of GM-CSF stimulation (Figure 2C). Our results indicate a decoupling of hyperactive ERK signaling from expansion and proliferation in NP^mut^ HSPCs.

Interestingly, despite the comparable expansion of lymphoid-primed MPP4 cells in Nras^G12D^ and NP^mut^ mice (Supplemental Figure 4E), the numbers of downstream common lymphoid progenitors (CLPs) (defined as Lin^–^IL-7Rα^+^Sca1^lo^cKit^lo^) were significantly increased in Nras^G12D^ but not NP^mut^ BM and SP compared with controls (Figure 2D). Moreover, moribund primary NP^mut^ mice showed decreased T and B lymphocytes (Supplemental Figure 6) and invisible thymi (Figure 2E), whereas age-matched Nras^G12D^ mice displayed normal lymphocyte compartments (Supplemental Figure 6) and a moderate increase in thymus weight (Figure 2E). Similarly, in NP^mut^ recipients, in which host-derived WT T cells significantly contributed to the T cell compartment, the thymus weight were greatly reduced and the T cell compartment shrank in SP and PB (Figure 2, F and G). Our data demonstrate an imbalanced myelopoiesis and lymphopoiesis in NP^mut^ HSPCs that may be induced by mutant p53 and oncogenic NRAS in a cell-autonomous manner and further enhanced via secondary cell nonautonomous mechanism(s).

### Mutant p53 and oncogenic NRAS synergistically establish a distinct NP^mut^ AML transcriptome.

**Figure 3:**
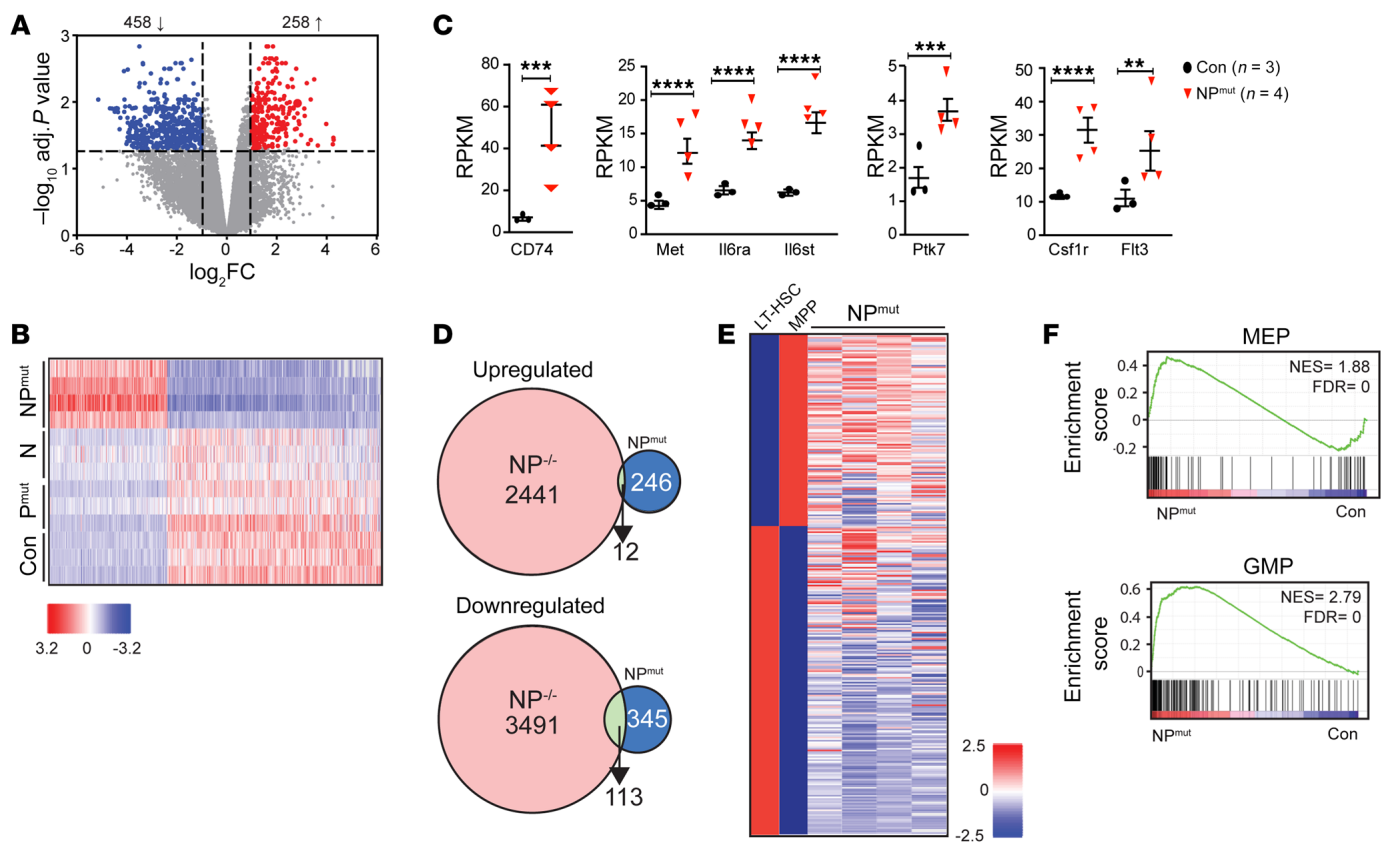
Mutant p53 and oncogenic NRAS synergize to establish the NP^mut^ AML transcriptome. Lin^–^cKit^+^ BM HSPCs were sorted from moribund NP^mut^ and age-matched control, p53^mut^, and Nras^G12D^ mice for RNA-Seq analysis. (**A**) Volcano plot of DEGs in NP^mut^ versus control HSPCs (upregulated genes are shown in red and downregulated genes in blue). (**B**) Heatmap of DEGs in control, p53^mut^ (P^mut^), Nras^G12D^ (N), and NP^mut^ HSPCs. (**C**) Quantification of transcriptional levels of RTKs. Results are presented as the mean ± SD. **P < 0.01, ***P < 0.001, and ****P < 0.0001. (**D**) Venn diagrams of overlapped DEGs in NP^–/–^ versus NP^mut^ HSPCs. (**E** and **F**) NP^mut^ HSPCs displayed a MPP gene signature (**E**) and partial signatures of MEPs and GMPs (**F**). NES, normalized enrichment score. (**C** and **F**) Wald tests within DESeq2 were conducted to assess differential gene expression between groups. *P* values from DEG analyses and GSEA were corrected for multiple testing using the Benjamini-Hochberg method.

To investigate how mutant p53 cooperates with oncogenic NRAS to promote leukemogenesis, we performed RNA-Seq using sorted Lin^–^cKit^+^ BM HSPCs from moribund NP^mut^ as well as pI-pC–treated, age-matched control, p53^mut^, and Nras^G12D^ mice. RNA-Seq analysis of NP^mut^ versus control HSPCs identified 716 differentially expressed genes (DEGs) (fold change ≥2, FDR/adjusted *P* < 0.05), with 258 and 458 genes significantly up- and downregulated, respectively (Figure 3A). The transcriptional levels of these DEGs in p53^mut^ and Nras^G12D^ HSPCs were indistinguishable from those in controls (Figure 3B), suggesting that mutant p53 and oncogenic NRAS synergistically established the aberrant NP^mut^ AML transcriptome.

We investigated potential mechanisms underlying ERK1/2 hyperactivation in NP^mut^ HSPCs. Consistent with the genotyping results (Figure 1B), we found that transcripts from both WT and oncogenic *Nras* alleles were expressed at similar levels and that *Nras* itself was not differentially expressed in NP^mut^ HSPCs compared with controls (data not shown). Among the established positive regulators of the ERK1/2 signaling pathway (*Sos1/2*, *Rasgrp1-4*, and *Ptpn11*), only *Rasgrp4*, encoding a RAS guanine nucleotide exchange factor, was moderately upregulated. However, *Rasgrp4* levels were almost undetectable (reads per kilobase per million mapped reads [RPKM] <1) (Supplemental Figure 7, A and B). Evaluation of established negative regulators (e.g., *Spry 1–4*, *Socs* family members, *Cbl*, *Dusp1*, and *Nf1*) revealed that *Dab2ip*, a RAS GTPase activating protein, was downregulated in NP^mut^ HSPCs (Supplemental Figure 7, C and D). Further examination of the 258 upregulated DEGs identified increased expression of several genes encoding receptor-like tyrosine kinases (RTKs) upstream of ERK1/2, including MET and the IL-6 receptor (Figure 3C). These findings suggest that overexpression of RTKs may contribute to the hyperactivation of MEK/ERK signaling in NP^mut^ HSPCs.

Consistent with the notion that the NP^mut^ AML transcriptome is mainly driven by the synergistic activities of mutant p53 and oncogenic NRAS, it showed minimal overlap with the NP^-/-^ AML transcriptome, which is predominantly driven by *p53* loss (14) (Figure 3D). Common upregulated genes shared between both AML transcriptomes were enriched for molecular signatures related to the RAS pathway (e.g., *Junb*), whereas common downregulated genes were enriched for NPM1-mutated or MLL1-driven AML-related gene sets (Supplemental Table 1). Using published data sets (31, 32), we previously showed that NP^–/–^ HSPCs gain partial HSC signature and largely retain the MEP signature (14). Consequently, NP^–/–^ MEPs, but not GMPs, are transformed into AML-initiating cells (14). By contrast, NP^mut^ HSPCs displayed a MPP gene signature (Figure 3E) and partial signatures of both MEPs and GMPs (Figure 3F). Not surprisingly, LSK and MPP2–4 cells sorted from approximately 6-week-old NP^mut^ mice could reestablish AML in 100% of the recipient mice, whereas NP^mut^ GMPs and MEPs only reinitiated AML in a fraction of recipients (Supplemental Table 2).

### Mutant p53 and oncogenic NRAS cooperatively dysregulate hematopoietic transcription factor networks and promote inflammation.

**Figure 4:**
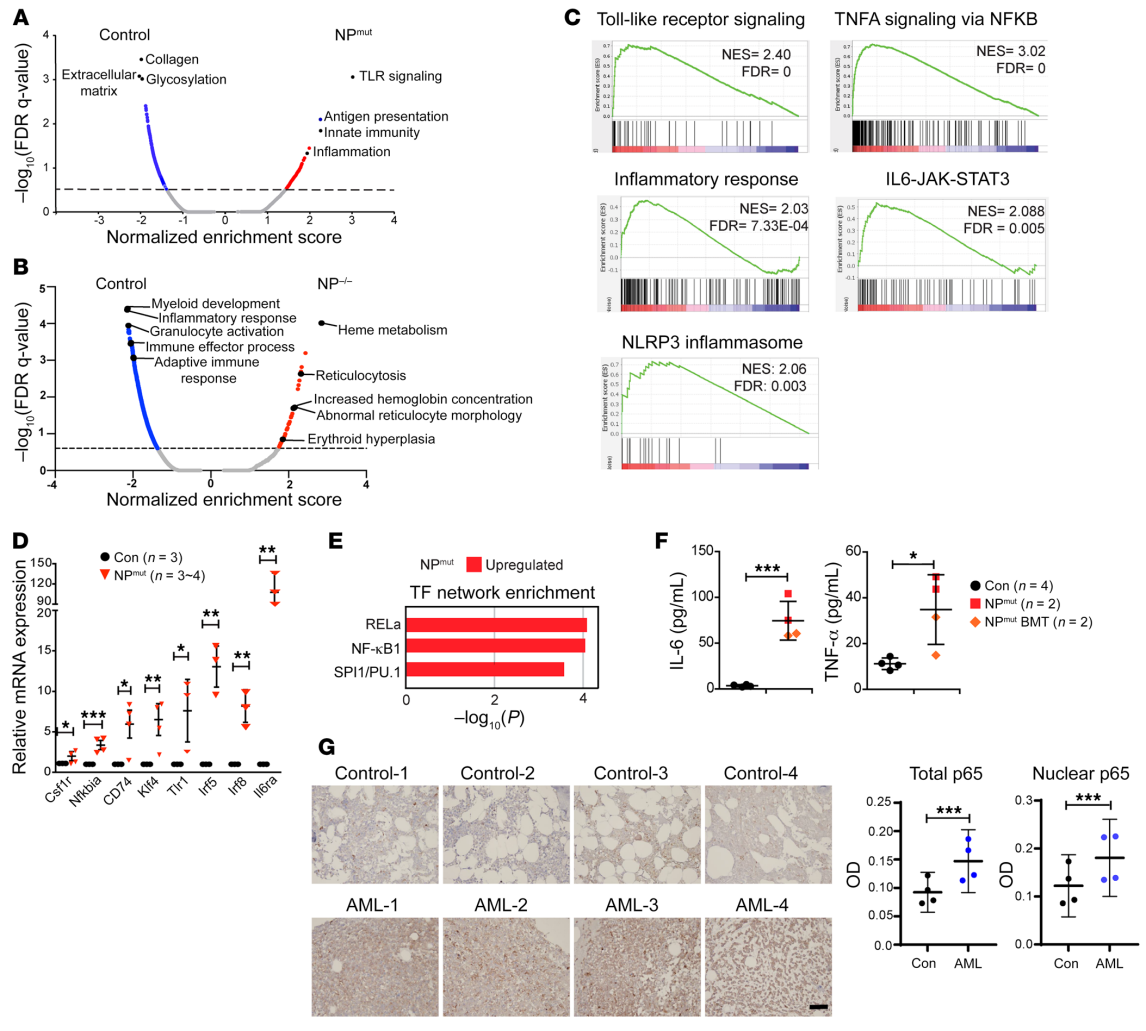
Upregulation of NF-κB in NP^mut^ HSPCs and *NRAS TP53* AML cells. (**A** and **B**) GSEA identified distinct pathways enriched in NP^mut^ (**A**) and NP^–/–^ (**B**) HSPCs. (**C**) Enrichment of inflammation-related pathways in NP^mut^ cells. (**D**) qRT-PCR validation of several inflammation-related genes. (**E**) Dysregulation of RELa, NF-κB1, and SPI1/PU.1 transcriptional networks in genes upregulated in NP^mut^ HSPCs. (**F**) Quantification of inflammatory cytokines in serum from primary and NP^mut^ recipient mice. (**G**) Immunohistochemical staining for NF-κB p65 on human *NRAS TP53* AML BM cores. Scale bar: 100 mm. The OD of total and nuclear p65 was quantified (see Supplemental Methods for details). (**D**, **F**, and **G**) Results are presented as the mean ± SD. *P < 0.05, **P < 0.01, and ***P < 0.001, by unpaired, 2-tailed Student’s *t* test (**D** and **F**) and 1-way ANOVA followed by Tukey’s post hoc test (**G**).

We performed gene set enrichment analysis (GSEA) comparing NP^mut^ with control HSPCs against the gene sets available in the Molecular Signatures Database (MSigDB) (33). Several gene sets related to inflammation and innate immunity were enriched in NP^mut^ HSPCs, whereas gene sets associated with extracellular matrix reorganization were predominantly enriched in control cells (Figure 4A). In contrast to the enrichment of erythroid differentiation pathways in NP^–/–^ HSPCs (Figure 4B), the TLR signaling pathway, TNF-α signaling via the NF-κB, inflammatory response, IL-6/JAK/STAT3 signaling, and the NLRP3 inflammasome were overrepresented in NP^mut^ cells (Figure 4C). In addition, many regulatory components of these pathways, such as *Csf1r*, *Nfkbia*, *CD74*, *Tlr1*, *Irf5/8*, and *Il6ra* were significantly upregulated in the NP^mut^ AML transcriptome, and their overexpression was validated using quantitative reverse transcription PCR (qRT-PCR) (Figure 4D). Consistent with the GSEA data, analysis via Metascape, an online tool that integrates information from several databases (e.g., Transcriptional Regulatory Relationships Unraveled by Sentence-based Text [TRRUST]) (34, 35), identified that transcriptional networks mediated by NF-κB pathway TFs (*Rela* and *Nfkb1*) and the myeloid/B lineage transcriptional regulator (PU.1) were enriched in upregulated genes in NP^mut^ HSPCs (Figure 4E). Because activation of the TLR/NF-κB signaling pathway often leads to overproduction of inflammatory cytokines and chemokines (36–38), we examined the levels of select inflammatory cytokines in the serum of primary NP^mut^ mice and NP^mut^ recipient mice using a multiplex ELISA (Figure 4F). This analysis revealed elevated levels of several inflammatory cytokines, including IL-6 and TNF-α, indicating systemic inflammation in NP^mut^ mice. To determine whether our result with NP^mut^ mice informs human AML, we performed immunohistochemical staining of NF-κB p65 on human specimens, including 4 control and 4 AML BM cores. Control BM biopsies were collected from patients with clinical histories of thrombocytopenia, monoclonal gammopathy of undetermined significance, or Hodgkin lymphoma, but who had a normal BM biopsy as assessed by a hematopathologist. AML BM cores were from patients with AML who had both *NRAS* and *TP53* mutations. Consistent with our mouse data, total and nuclear p65 levels were upregulated in AML blast cells versus control BM cells (Figure 4G).

**Figure 5:**
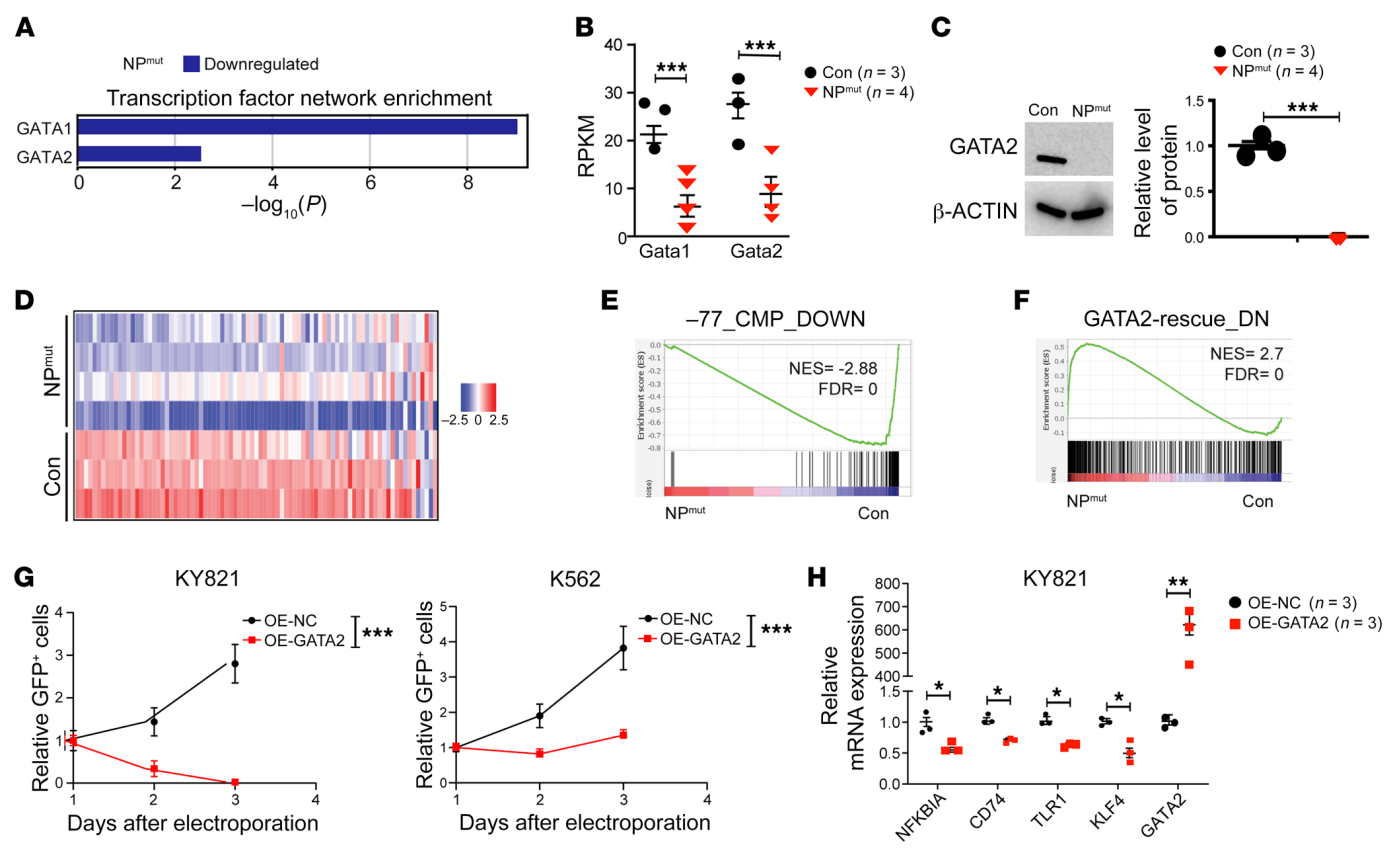
GATA2 regulates transcriptional levels of inflammation-related genes and survival of mouse and human NP^mut^ cells. (**A**) Dysregulation of GATA1 and GATA2 transcriptional networks in genes downregulated in NP^mut^ HSPCs. (**B**) Quantification of *Gata1* and *Gata2* transcriptional levels. (**C**) Western blot analysis of GATA2 protein levels in control and NP^mut^ HSPCs. (**D**) Heatmap of genes downregulated in *Gata2* enhancer –77^–/–^ versus control fetal liver MPs. (**E** and **F**) Genes downregulated in –77^–/–^ MPs were enriched in control HSPCs (**E**), whereas genes downregulated upon GATA2 reexpression were enriched in NP^mut^ HSPCs (**F**). (**G** and **H**) Human NP^mut^ KY821 AML cells were electroporated with MSCV-GFP (OE-NC) or MSCV-GATA2-GFP (OE-GATA2) DNA. (**G**) Quantification of transduced KY821 and K562 cells in culture. (**H**) Quantification of *GATA2* and inflammation-related genes via qRT-PCR 48 hours after electroporation. (**B**, **C**, **G**, and **H**) Results are presented as the mean ± SD. (**B**, **E**, and **F**) Wald tests within DESeq2 were conducted to assess differential gene expression between groups. *P* values from differential gene expression analyses and GSEA were corrected for multiple testing using the Benjamini-Hochberg method. *P < 0.05, **P < 0.01, and ***P < 0.001, by unpaired, 2-tailed Student’s *t* test (**C** and **H**) and 2-way ANOVA followed by Tukey’s post hoc test (**G**).

Our Metascape analysis of downregulated genes in NP^mut^ HSPCs revealed enrichment for GATA1- and GATA2-linked transcriptional networks (Figure 5A), which included downregulated expression of *Gata1* and *Gata2* themselves (Figure 5B). GATA2 downregulation in NP^mut^ HSPCs was further validated using Western blot analysis (Figure 5C). Since GATA2 regulates *Gata1* expression (39), our data indicate a loss of GATA2 TF activity. Accordingly, the genes downregulated in GATA2-deficient CMP/GMP cells from *Gata2* enhancer –77^–/–^ fetal liver (40) were also downregulated in NP^mut^ HSPCs (Figure 5, D and E). More important, analogous to what we observed in NP^mut^ HSPCs, *Gata2* downregulation in fetal liver MPs resulted in the upregulation of TLR and IFN pathways, with enrichment of genes representing a spectrum of inflammatory mechanisms (41, 42). In a rescue assay, in which the capacity of GATA2 to regulate endogenous target genes was quantified using RNA-Seq and qRT-PCR (41, 43–45), GATA2 reexpression downregulated the expression of inflammation-related genes in *–77^–/–^* fetal liver MPs (GATA2-rescue_DN gene set). Although this gene set was not included in the databases we previously used, it was significantly enriched in NP^mut^ HSPCs (Figure 5F). We conducted a similar GATA2 reexpression analysis in Lin^–^cKit^+^ HSPCs, which were sorted from control and NP^mut^ BM, cultured with cytokines in RetroNectin-coated wells, and infected with MSCV empty vector or a MSCV GATA2 construct as described previously (46). GATA2 reexpression led to rapid cell death in NP^mut^ HSPCs, precluding downstream analyses. Thus, we induced expression of GATA2 in human NP^mut^ KY821 AML cells (20) and K562 cells with WT *NRAS* and *TP53* loss via electroporation. The number of GATA2-expressing KY821 cells quickly declined in culture (Figure 5G), and GATA2 downregulated the expression of inflammatory genes (Figure 5H). The growth-inhibitory effect of GATA2 was also observed in K562 cells, but to a lesser degree (Figure 5G). Our data suggest that downregulation of the GATA2 transcriptional network contributed to pathologic inflammation in NP^mut^ mice.

### Inhibition of MEK and NF-κB signaling attenuates NP^mut^ cell growth in vitro and in vivo.

**Figure 6:**
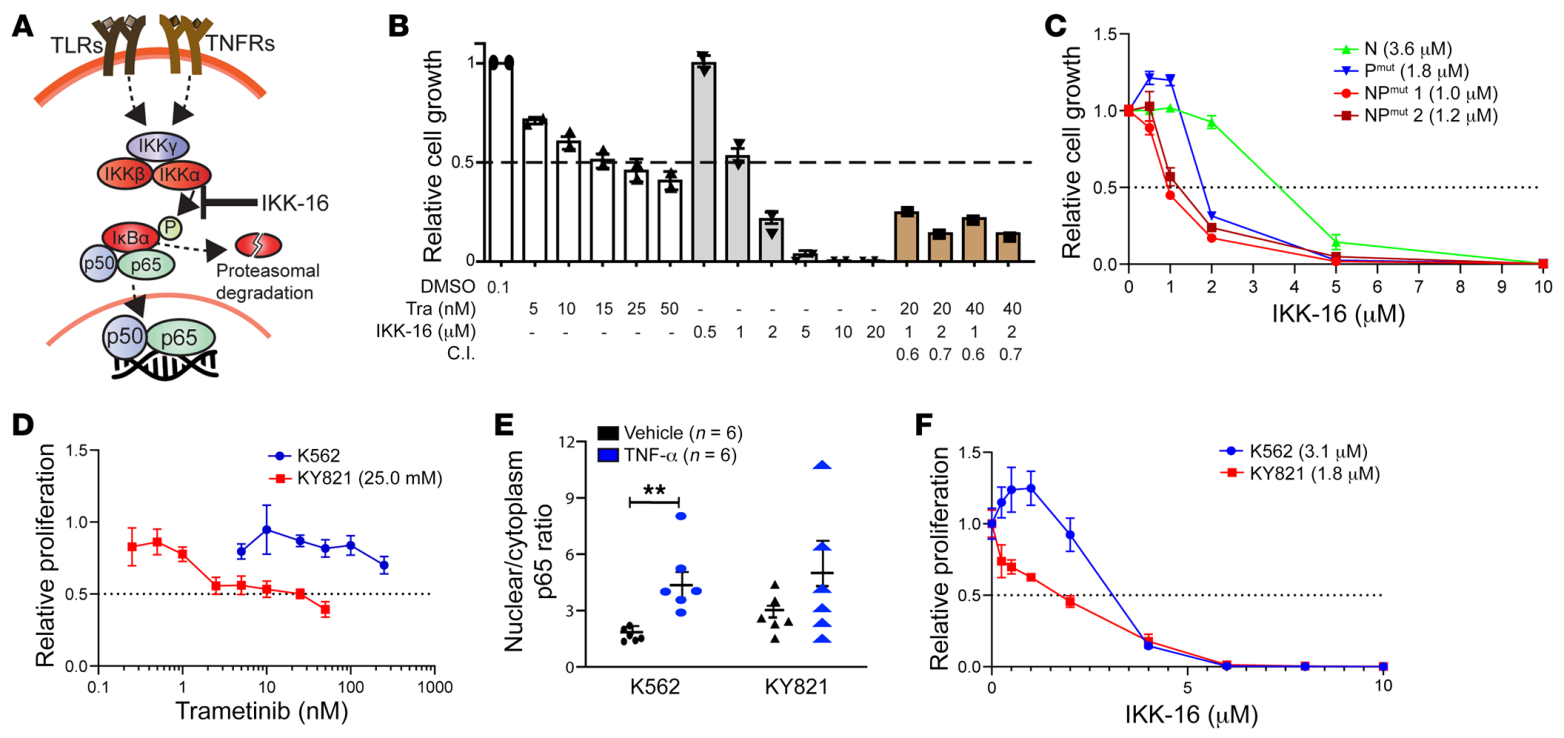
Inhibition of MEK and NF-κB signaling blocks the growth of mouse and human NP^mut^ leukemia cells in vitro. (**A**) Schematic illustration of NF-κB signaling. (**B**) Quantification of mouse NP^mut^ cell growth using the CellTiter-Glo assay. The Combination Index (C.I.) was calculated. A Combination Index of less than 1 indicates synergism. (**C**) IKK-16 dose-response curves of BM cells from moribund Nras^G12D^ (N), p53^mut^ (P^mut^), and NP^mut^ mice. (**D**) Tra dose-response curves of human K562 and KY821 leukemia cell lines. (**E**) Quantification of nuclear versus cytoplasmic NF-κB p65 localization in K562 and KY821 cell lines. **(F**) IKK-16 dose-response curves for K562 and KY821 cell lines. (**B**–**F**) Results are presented as the mean ± SD. **P < 0.01, by 1-way ANOVA followed by Tukey’s post hoc test (**E**).

Upon TLR and TNF-α receptor activation, the IκB kinase (IKK) complex is activated and phosphorylates the inhibitory protein IκBα, leading to its proteasome-mediated degradation and subsequent nuclear localization of NF-κB TFs (Figure 6A) (47). IKK-16, a selective IKK inhibitor (48), is a well-established tool compound used to inhibit NF-κB activation. To determine whether blocking hyperactivated MEK/ERK and/or NF-κB signaling inhibits NP^mut^ AML cell growth in vitro, we cultured mouse NP^mut^-AML cells in the presence of the FDA-approved MEK inhibitor trametinib (Tra) (49) and/or IKK-16. Both drugs killed NP^mut^ AML cells alone in a dose-dependent manner with the IC_50_ at approximately 15 nM and approximately 1 μM, respectively (Figure 6B). Combined Tra and IKK-16 inhibited NP^mut^-AML cell growth more effectively than did a single agent alone (Combination Index <1 indicates synergism). By contrast, BM cells isolated from moribund p53^mut^ mice (IC_50_: 1.8 μM) or Nras^G12D^ mice (IC_50_: 3.6 μM) were less sensitive to IKK-16 (Figure 6C).

Consistent with our mouse results, NP^mut^ KY821 AML cells showed similar sensitivity to Tra (IC_50_: 25 nM), whereas K562 cells were resistant to Tra (IC_50_ >200 nM) (Figure 6D). To determine whether the NF-κB pathway is elevated in human NP^mut^ AML cells, we quantified the nuclear versus cytosolic localization of NF-κB p65 in K562 and KY821 cells with or without 3 ng/mL TNF-α stimulation using a confocal immunofluorescence microscopy–based method similar to that described in a previous publication (50) (Figure 6E). KY821 cells had a higher nuclear/cytosolic p65 ratio than did K562 cells under unstimulated conditions, indicating an elevated basal activation of NF-κB signaling in KY821 cells. Upon TNF-α stimulation, nuclear localization of NF-κB was significantly increased in K562 cells, while an increase was trending but statistically insignificant in KY821 cells, probably due to high and potentially saturated basal NF-κB activity in these cells. As expected, KY821 cells were more sensitive to IKK-16 treatment than were K562 cells (IC_50_: 1.8 μM vs. 3.1 μM) (Figure 6F). Our results indicate that NP^mut^ AML cells were sensitive to MEK and NF-κB inhibition in vitro.

**Figure 7:**
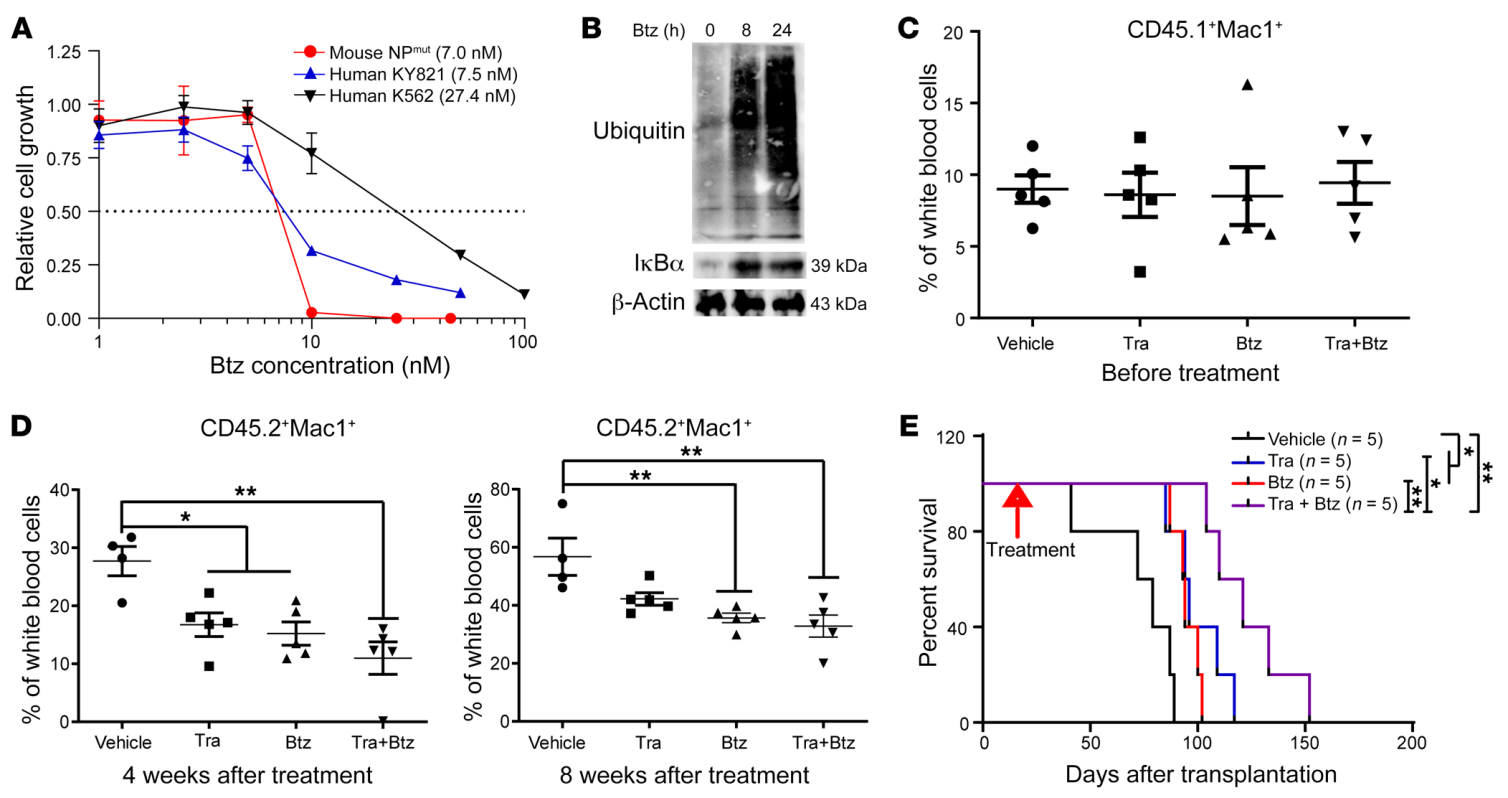
Combined MEK and proteasome inhibitors ameliorate AML burden and prolong the survival of NP^mut^ mice. (**A**) Btz dose-response curves of mouse NP^mut^ leukemia cells and human K562 and KY821 cell lines. (**B**) Western blot analysis of ubiquitin and IκBα in human KY821 cells treated with 5 nM Btz. (**C**–**E**) NP^mut^ cells were transplanted into sublethally irradiated CD45.1^+^ recipients. Once AML was established, the recipient mice were treated with vehicle, Tra, Btz, or combined Tra and Btz until moribund. (**C** and **D**) Quantification of leukemia burden before (**C**) and after (**D**) drug treatment. (**C** and **D**) Results are presented as the mean ± SD. (**E**) Kaplan-Meier survival curves for different treatment cohorts. *P < 0.05 and **P < 0.01, by 1-way ANOVA followed by Tukey’s post hoc test (**C** and **D**) and log-rank test followed by Benjamini-Hochberg multiple-comparison analysis (**E**).

We did not pursue any in vivo studies with IKK-16, given the established toxicities of IKK inhibitors (51). By contrast, we discovered that NP^mut^ HSPCs overexpressed CD74 (Figure 4C), whose increased expression correlates to the complete remission in patients with AML treated with the combined proteasome inhibitor bortezomib (Btz) and induction chemotherapy (52), as well as to Btz sensitivity in patients with multiple myeloma (53). Therefore, we treated NP^mut^ leukemia cells with Btz in vitro. Human myeloma cell lines with intermediate/high sensitivity to Btz typically have an IC_50_ of less than 10 nM (54). Both human and mouse NP^mut^ leukemia cells were more sensitive to Btz (IC_50_: ~7–8 nM) than were K562 cells (IC_50_: 27.4 nM) (Figure 7A). Consistent with the known action of Btz in inhibiting the NF-κB pathway (55, 56), Btz-treated KY821 cells showed accumulation of ubiquitinylated proteins and stabilization of IκBα, the inhibitory protein of NF-κB p65 (Figure 7B). Our data suggest that the antitumor effect of Btz was mediated, at least partially, through inhibition of NF-κB p65 activity.

We further examined Btz effects in vivo. NP^mut^ leukemia cells were transplanted into sublethally irradiated mice. Upon establishment of AML, the recipient mice were divided into 4 groups with comparable leukemia cell burdens and treated with vehicle, Tra, Btz, or combined Tra and Btz (Figure 7C). Tra alone and Btz alone lowered the leukemia burden (Figure 7D) and prolonged the survival of NP^mut^ mice (Figure 7E). Combination treatment further potentiated the survival benefits with the use of a single agent alone (Figure 7E). To determine the mechanisms of drug treatment, we conducted an independent experiment and sacrificed vehicle-treated moribund mice along with Btz- or combination drug–treated mice, which carried the average leukemia burden in their corresponding groups. Donor-derived leukemia cells were flow sorted from BM, and the transcript levels of inflammation-related genes were quantified using qRT-PCR. This analysis revealed that the leukemia suppression effects of Btz and combination treatment were associated with reduced expression of inflammation-related genes (Supplemental Figure 8).

## Discussion

We discovered that mutant p53 and oncogenic NRAS synergized to promote inflammation and AML via distinct mechanisms from single mutants and from NP^–/–^. Systemic inflammation in NP^mut^ mice was demonstrated in several assays. First, we found that NP^mut^ mice were hypersensitive to pI-pC injection and died within a few days after the first pI-pC injection (Supplemental Figure 1B), consistent with inflammation-induced acute lethality. Second, RNA-Seq analysis identified the upregulation of inflammation-related gene signatures and overexpression of inflammation-related genes in NP^mut^ HSPCs (Figure 4, A, C, and D). Third, we detected elevated levels of multiple inflammatory cytokines in NP^mut^ serum samples (Figure 4F). Our finding is consistent with prior literature showing that inflammation is involved in de novo AML progression, chemoresistance, and suppression of normal hematopoiesis (57, 58).

Despite the marked hyperactivation of MEK/ERK signaling in NP^mut^ HSPCs (Figure 2C), we did not detect further expansion of NP^mut^ HSPC compartments compared with those in Nras^G12D^ mice (Figure 2A). Moreover, NP^mut^ HSPCs displayed cell-cycle profiles comparable to those in control HSPCs (Supplemental Figures 4 and 5). This is in sharp contrast to what we and others reported in multiple oncogenic NRAS and KRAS models, in which stronger MEK/ERK signaling leads to greater expansion and hyperproliferation of HSPCs (26, 30, 59–63). It is possible that the inflammatory state of NP^mut^ mice leads to decoupling of hyperactive ERK signaling from HSPC expansion and proliferation. In support of this model, we found that KLF family genes, such as *Klf4*, were upregulated in NP^mut^ HSPCs (Figure 4D). KLF4 was initially identified as a TF associated with cell-growth arrest (64) and is important for promoting quiescent transcriptional programs and cell survival in endothelial cells and myeloid cells under inflammatory conditions (65). Furthermore, our analyses revealed upregulation of the PU.1-mediated transcriptional network (Figure 4E), which is known to enforce quiescence and limit HSPC expansion during inflammatory stress (66).

Unlike Nras^G12D^ HSPCs with balanced expansion in myeloid and lymphoid compartments, NP^mut^ HSPCs showed imbalanced myelopoiesis and lymphopoiesis (Figure 2A), to which both cell-autonomous and cell-nonautonomous mechanisms may contribute. We previously reported that GATA2 downregulation in HSPCs reduces lymphoid progenitors and the reconstitution of T cells in comparison with WT HSPCs (67). Consistently in this study, we found that decreased GATA2 expression in NP^mut^ HSPCs was associated with lymphopenia (Figure 5), suggesting that mutant p53 and oncogenic NRAS cooperated to downregulate GATA2 and regulate hematopoiesis in a cell-autonomous manner. Since the immune checkpoint pathways were largely normal in NP^mut^ mice (data not shown), we believe that the reduced T cell compartment in NP^mut^-AML recipients did not result from a suppressive immune microenvironment, as we had previously described in *Nras^G12D/+^* *Asxl1^–/–^* mice (10). By contrast, increased systemic inflammation has been shown to promote myelopoiesis at the expense of lymphopoiesis (68–72). Similarly, NP^mut^ recipients exhibited thymic dystrophy (Figure 2F) and reduced T cell compartments (Figure 2G), which included both WT and NP^mut^ T cells. Therefore, inflammation in NP^mut^ mice may result in reduced T cells through a cell-nonautonomous mechanism.

GATA2-mediated inhibitory mechanisms in NP^mut^ AML cells are distinct from those in acute promyelocytic leukemia (73). GATA2 restricts innate immune pathways and inflammation-related pathways in fetal liver MPs (41, 42, 44, 45) and in NP^mut^ HSPCs (Figure 5). Under these drastically different settings, GATA2 downregulation was associated with elevated levels of innate immune signaling and inflammatory gene transcripts, while GATA2 reexpression restored their normal expression pattern. A subset of these genes is occupied by PU.1 (74–76). When GATA2 levels decline, increased PU.1 activity promotes the upregulation of innate immune gene transcription (44). *Gata2* can be transcriptionally regulated through GATA1-mediated repression (77) and GATA2-mediated positive autoregulation (43, 78). SCL/TAL1 activates *Gata2* transcription, in part through occupation of the *Gata2* +9.5 enhancer (79). In addition, LSD-1 suppresses *Gata2* transcription in *TET2^mut^* AML (80). It is likely, therefore, that multiple mechanisms contribute to *Gata2* downregulation in NP^mut^ AML cells.

Not surprisingly, the transcriptional network of NF-κB TFs, downstream of innate immune pathways and inflammation-related pathways, was enriched in upregulated genes in NP^mut^ HSPCs (Figure 4E). Similarly, NF-κB p65 was overexpressed in human *NRAS* *TP53* AML cells (Figure 4G). These results suggest that NP^mut^ AML cells may be sensitive to NF-κB inhibition. Indeed, mouse and human NP^mut^ AML cells were sensitive to IKK-16 in vitro (Figure 6). Btz downregulated inflammation-related gene expression and prolonged the survival of NP^mut^ mice in vivo (Figure 7 and Supplemental Figure 8). Given the wide-ranging activities of Btz, it is possible that Btz functioned through both NF-κB–dependent and –independent mechanisms.

We demonstrate that NP^mut^ induced phenotypic, cellular, and molecular changes distinct from NP^–/–^. At the phenotypic level, NP^–/–^ mice developed a mixed AML and T cell lymphoma/leukemia (14), while NP^mut^ mice rapidly developed an AML-like disease with decreased T cell numbers (Figures 1 and 2). At the cellular level, NP^–/–^ MPs showed further expansion and hyperproliferation over Nras^G12D^ cells, whereas NP^mut^ MPs displayed a moderate reduction compared with Nras^G12D^ MPs and comparable cell-cycle profiles to control MPs (Supplemental Figure 5). NP^–/–^ HSPCs gained a partial HSC signature and largely retained their MEP signature. Consequently, NP^–/–^ MEPs, but not GMPs, were transformed into AML-initiating cells. By contrast, NP^mut^ HSPCs displayed a MPP gene signature (Figure 3E) and partial signatures of both MEPs and GMPs (Figure 3F). Not surprisingly, MPP2–4 were fully transformed, whereas MEPs and GMPs were partially transformed into AML-initiating cells (Supplemental Table 2). At the molecular level, the NP^–/–^ AML transcriptome was predominantly regulated by *p53* loss, whereas the NP^mut^ AML transcriptome was driven by the synergistic interaction between mutant p53 and oncogenic NRAS signaling (Figure 3B) and had minimal overlap with the NP^–/–^ AML transcriptome (Figure 3D). The predominant inflammatory gene signature seen in NP^mut^ AML was therefore absent in NP^–/–^ AML. Consistent with this finding, hyperactivation of ERK signaling in NP^–/–^ HSPCs resulted from homozygosity of the *Nras^G12D^* allele and Nras protein overexpression, whereas ERK hyperactivation in NP^mut^ HSPCs was mainly mediated by overexpression of RTKs (Figure 3C). Taken together with the previous report (20), *p53^R172H^* had GOF in *p53^R172H/–^* AML and in the context of *Nras^G12D^*-driven leukemogenesis.

Compared with the prevalent p53^R248W^ mutant, p53^R172H^ led to increased BM reconstitution (Supplemental Figure 9A) through a distinct mechanism, as the expression levels of several important DEGs in p53^R248W^ HSPCs (19) were comparable between p53^R172H^ and control cells (Supplemental Figure 9B). In addition, the known mutant p53–interacting TFs identified in solid tumor cells or p53^R172H/–^ AML cells were not expressed at significant levels in NP^mut^ HSPCs, nor were the known mutant p53 target genes dysregulated in NP^mut^ HSPCs (20, 81). These data suggest that cell type– and/or genotype-specific TF(s) may interact with mutant p53 to promote tumorigenesis. It is likely that p53^mut^ gains novel interactions with TF(s) downstream of hyperactivated ERK signaling to promote inflammation and NP^mut^ AML. Given these results, we expect that p53^R172H^ would display distinct properties in the context of other mutations found in AML.

## Methods

### Mice.

Mouse lines were maintained on a pure C57BL/6J genetic background (>N10). Genotyping of *Nras^LSL G12D/+^*, *p53^LSL-R172H/+^* (stock 01XAF, NCI), *Mx1-Cre*, and *Vav-Cre* mice was done as previously described (22, 26, 59, 82). CD45.1^+^ congenic C57BL/6J recipient mice were purchased from The Jackson Laboratory (stock 002014). To induce Mx1-Cre expression, approximately 6-week-old mice were injected i.p. with 100 μg pI-pC (GE Healthcare) every other day for 3 cycles. The day of the first pI-pC injection was defined as day 1. All experiments were performed on day 12 or at the moribund stage.

### Statistics.

All results are presented in dot plots with the mean ± SD. All in vitro studies were performed at least 3 times, with 2–3 technical replicates for each condition. Results from 1 representative experiment are shown. A log-rank test followed by a Benjamini-Hochberg multiple-comparison analysis was used to compare Kaplan-Meier survival curves. An unpaired, 2-tailed Student’s *t* test was used to compare 2 data sets unless otherwise specified. A 1-way ANOVA followed by Tukey’s post hoc test for multiple comparisons was used to compare more than 2 data sets with 1 variable. A 2-way ANOVA followed by Tukey’s post hoc test for multiple comparisons was used to compare more than 2 data sets with 2 variables, whereas a 2-way ANOVA followed by Bonferroni’s multiple-comparison test was used to compare 2 data sets with 2 variables. *P* values and adjusted *P* values are indicated in the figure legends. A *P* value of 0.05 or less was considered significant. ImageJ software (NIH) was used to quantify protein levels by densitometry. Data were graphed and analyzed using GraphPad Prism 7.0 (GraphPad Software). The synergy score was calculated using the Combination Index to characterize the strength of synergistic interaction between 2 drugs.

### Study approval.

All animal experiments were conducted in accordance with the NIH’s *Guide for the Care and Use of Laboratory Animals* (National Academies Press, 2011) and approved by the IACUC of the University of Wisconsin–Madison. The program is accredited by the Association for Assessment and Accreditation of Laboratory Animal Care (protocol M005328). All human samples were obtained from the University of Wisconsin Hospitals and Clinics with IRB approval (protocol 2014-0904).

### Data availability.

All data in this study are presented in the article and supplemental materials. All materials are available upon request through a material transfer agreement; inquiries should be directed to the corresponding author. Values for all data points in graphs are reported in the Supporting Data Values file. RNA-Seq data were deposited in the NCBI’s Gene Expression Omnibus (GEO) database (GEO GSE243642).

Additional methods are described in Supplemental Methods.

## Author contributions

AR, YF, and MBG designed and performed experiments and wrote and reviewed the manuscript. EAR conducted histopathological analysis and reviewed the manuscript. TK, MHL, YZ, XG, and SM provided technical support and reviewed the manuscript. DRM, MMJ, KVGN, DY, VLT, EP, and EHB provided material support and reviewed the manuscript. JZ supervised the study, designed experiments, and wrote and reviewed the manuscript.

## Supplementary Material

Supplemental data

Supplemental table 1

Supporting data values

## Version 1 10/17/2023

In-Press Preview

## Version 2 12/15/2023

Electronic publication
