# Supplementary material for: A gain-of-function p53 mutant synergizes with oncogenic NRAS to promote acute myeloid leukemia in mice
Source: J Clin Invest. 2023 Dec 15;133(24):e173116. doi: 10.1172/JCI173116 (PMC10721149; doi:10.1172/JCI173116)
Supplement: Supplemental data [file jci-133-173116-s238.pdf]

Supplemental Information

Supplemental Methods

Complete blood count and histopathology

Complete blood count analysis was performed using a Hemavet 950FS (Drew Scientific). Mouse tissues were fixed in 10% neutral buffered formalin (Sigma-Aldrich) and further processed at the UWCCC Histology Lab.

Flow cytometric analysis of hematopoietic tissues

For lineage analysis of bone morrow, spleen and peripheral blood, flow cytometric analyses were performed as previously described [1]. HSCs, MPPs, LSK and MPs in bone marrow and spleen were analyzed as previously described [2]. LT-HSC, ST-HSC, MPP2-4 in bone marrow and spleen were analyzed as previously described [3]. Stained cells were analyzed on a FACS Fortessa or LSRII (BD Biosciences). Directly conjugated or biotin conjugated antibodies against the following surface antigens were purchased from eBioscience: CD45.1 (A20), CD45.2 (104), Mac-1 (M1/70), Gr-1 (RB6-8C5), CD3 (145-2C11), CD4 (RM4-5), CD8 (53-6.7), CD19 (eBio1D3), Thy1.2 (53-2.1), TER119 (TER-119), B220 (RA3-6B2), IgM (eB121-15F9), IL-7Rα (B12-1), CD41 (eBioMWReg30), CD48 (HM48-1), Sca1 (D7), c-Kit (2B8), and CD34 (clone RAM34). FcγRII/III (2.4G2) was purchased from BD Biosciences. CD150 (TC15-12F12.2) was purchased from Biolegend.

Cell cycle analysis

Cell cycle analysis was performed essentially as described [2]. Fixed cells were simultaneously stained with PECy7-conjugated antibodies against CD41, CD48, B220,

TER119 and Gr1, PE-CD150, APC-c-Kit, PerCPCy5.5-Sca1, FITC-Ki67 (BD Biosciences), and DAPI (Invitrogen). The stained cells were analyzed on a Fortessa or LSRII (BD Biosciences).

Colony assay and replating assay

5 x 10^4 bone marrow cells were plated in duplicate in semisolid medium MethoCult M3234 (StemCell Technologies) supplemented with mGM-CSF or mIL-3 (Peprotech, Rocky Hill, NJ) according to the manufacture's protocol. The colonies were counted after 7 to 10 days in culture. Then colonies were harvested to repeat the same procedure for serial replating.

Flow cytometric analysis of phospho-ERK1/2

Phosphorylated ERK1/2 was analyzed in defined Lin-/low c-Kit+ and Lin-/low c-Kit- cells essentially as previously described [4]. Surface proteins were detected with FITC-conjugated antibodies (BD Biosciences unless specified) against B220 (6B2), Gr-1 (RB6-8C5), CD3 (17A2, Biolegend), CD4 (RM4-5), CD8 (53-6.7), and TER119, and PE-conjugated anti-CD117/c-Kit antibody (eBiosciences). p-ERK1/2 was detected by a primary antibody against p-ERK (Thr202/Tyr204; Cell signaling Technology) followed by APC conjugated donkey anti-rabbit F(ab’)2 fragment (Jackson ImmunoResearch).

Murine bone marrow transplantation

1 x 10^6 total bone marrow cells ($CD45.2^+$) were mixed with same number of congenic bone marrow cells ($CD45.1^+$) and injected into individual lethally irradiated mice (8.5 Gy using an X-Rad 320 irradiator, Precision X-Ray) as previously described [5]. MEPs and GMPs were sorted using a FACS AriaII (BD Biosciences) as described [6]. Purified MEPs or GMPs

($CD45.2^{+}$) were transplanted with 2 x 10^5 whole bone marrow cells ($CD45.1^{+}$) into individual lethally irradiated mice. At the moribund stage, 1 x 10^6 bone marrow or spleen cells from recipients with AML were transplanted into individual sublethally irradiated mice (4.0 Gy) as shown in Fig. 1H.

RNA-Seq and data analysis

Total RNAs were isolated from 50,000 sorted Lin^{-} $cKit^{+}$ bone marrow cells of age-matched control (n=3), $p53^{R172H/+}$ (n=3), $Nras^{G12D/+}$ (n=3) and moribund NP^{mut} (n=4) mice using RNeasy Micro Kit (Qiagen). RNA-Seq libraries were prepared using SMARTer® Stranded Total RNA-Seq Kit v1 - Pico Input Mammalian (Takara Bio USA/Clontech). Sequencing was performed on an Illumina HiSeq 4000 system at the NUSeq Core facility. Reads from each sample were aligned to the Mus musculus GRCm38.p6 genome using STAR v2.6.1d 15. During alignment, the first three nucleotides (the switching oligonucleotides) were clipped from each read as recommended by Takara Bio USA/Clontech. Reads that aligned to the sense strand of genes within the Gencode release M24 genome annotation were then counted. Read counts for each sample were then imported into R v3.6.1 (https://www.R-project.org/) and normalized using DESeq2 v1.26.0 16. Wald tests within DESeq2 were conducted to assess differential gene expression between groups and the ashr method was used to shrink log2 fold-change values 17. Pre-ranked GSEA was conducted using clusterProfiler v3.14.3 18 using the Wald test statistics as the ranking values and the fgsea method. Gene sets from the Broad Institute's Molecular Signatures Database sets were used. P values from differential gene expression analyses and GSEA were corrected for multiple testing using the Benjamini-Hochberg method.

Quantification of inflammatory cytokines

Serum was collected from 8-weeks old primary NP^{mut}, age matched control, and NP^{mut} recipient mice (8 weeks post transplantation) and diluted 3-fold before assaying with MSD Cytokine Assays Proinflammatory Panel 1 V-Plex (mouse) kits (Meso Scale Discovery, Cat # K15048D). A panel of 10 pro-inflammatory cytokines were measured with the diluted serum sample by first pre-incubating with detection antibodies conjugated with electro chemiluminescent labels, then adding to a plate pre-coated with capture antibodies on independent spots and read on an MSD MESO QUICKPLEX SQ 120 multiplex cytokine plate reader to quantify cytokine levels.

Secondary bone marrow transplantation and drug treatment

Sub-lethally irradiated (4.0 Gy using an X-Rad 320 irradiator, Precision X-Ray) $CD45.1^+$ mice were transplanted with $0.5x10^6$ BM cells from moribund NP^{mut} primary recipients with AML phenotype. For treatment with NFκB and MEK inhibitors, secondary recipients were randomly separated into four groups at 2 weeks after transplantation and treated with vehicle, bortezomib (0.5mg/kg, every Monday and Thursday), trametinib (0.5mg/kg, once daily), or combined bortezomib and trametinib via oral gavage until the moribund stage.

Cell culture and drug treatment

Human KY821 cell line was purchased from Japanese Collection of Research Bioresources Cell Bank (Cat# JCRB0105, Lot# 11022006) and cultured in RPMI 1640 medium (GIBCO,

Cat# 11875-119) supplemented with 20% FBS (GIBCO, Cat# 16000-044). K562 cells were cultured in RPMI 1640 medium supplemented with 10% FBS. Cells were maintained at 37℃ in the presence of 5% CO_2. Bone marrow cells from moribund NP^{mut} mice with AML were cultured in SFEM (Stem Cell Technologies) with 10% FBS (GIBCO), 0.2ng/ml mGM-CSF (PeproTech, Cat#250-05). Cells were seeded at 2×10^5/ml in triplicate in 96-well plates in the presence of DMSO, various concentrations of IKK-16 (Selleckchem, Cat#S2882), trametinib (Selleckchem, Cat#S2673), or bortezomib (Selleckchem, Cat#S1013). After 3 days (human cell lines) or 5 days (mouse cells) in culture, cell viability was determined using the CellTiter Glo Assay (Promega, Cat#G7570) according to the manufacturer's instructions.

Western blot analyses

KY821 Cells were treated with 5nM bortezomib for 0-24 h before collection for Western blot analysis. Lin^- $c\text{-}Kit^+$ BM cells were flow sorted from moribund NP^{mut} mice with AML and age-matched control mice. Cells were washed with PBS and immediately lysed on ice for 30 min in RIPA Lysis Buffer (Invitrogen, Cat# 89901) and 1% PMSF Protease Inhibitor (Invitrogen, Cat# 36978). Cellular debris was removed by centrifugation. Lysates were heated at 95 °C for 5 min, separated on a NuPAGE™ 4 to 12%, Bis-Tris, 1.0–1.5 mm, Mini Protein Gels (Invitrogen, Cat# NP0335BOX), and transferred to Nitrocellulose Blotting membrane (GE Healthcare, Cat# 10600007). Membranes were blocked for 1 h at room temperature (RT) in blocking buffer (2% milk in PBS + 0.05% Tween-20). Primary antibodies were diluted in 0.2% milk in PBS + 0.05% Tween-20 and applied to the membrane overnight at 4 °C. HRP-conjugated secondary antibodies were diluted in 0.2%

milk in PBS + 0.05% Tween 20 and applied to the membrane for 1 h at RT. After washing in PBS + 0.05% Tween-20, clarity-enhanced chemiluminescence substrate (Invitrogen) was added to the membrane according to the manufacturer's instructions. Membranes were imaged using the Image Lab (Bio-Rad) and analyzed using Image Lab software. The following primary antibodies were used: anti-IκBα (Cell Signaling Technology, Cat# 4814, 1:1000), anti-Ubiquitin (Santa Cruz Biotechnology, Cat# sc-8017, 1:1000), and anti-β-Actin (Cell Signaling Technology, Cat# 8457S, 1:1000). Anti-GATA2 antibody was a custom produced mouse IgG monoclonal (clone 7A6) developed against human GATA2 (amino acids 1-204) and utilized at 1:1000 for western blotting (see upcoming manuscript by Robbins and Matson, in preparation, for additional details and validation studies). HRP conjugated goat anti-mouse secondary antibody was purchased from Invitrogen (Cat# 31430, 1:5000). HRP conjugated goat anti-rabbit secondary antibody was purchased from Jackson ImmunoResearch (Cat# 111-035-003, 1:5000).

qRT-PCR analysis of gene transcriptional levels

Lin^- $c\text{-}Kit^+$ cells were flow sorted from moribund NP^{mut} mice with AML and age-matched control mice. Total RNAs were extracted using RNeasy Micro Kit (Qiagen). cDNAs were synthesized using iScript™ Reverse Transcription Kit (Bio-Rad). Real-time PCR reactions were performed on a CFX96 Real-Time System (Bio-Rad) using the primers (Integrated DNA Technologies, Inc) specific for the following mouse genes: Csf1r (F: 5'-CAGTTCAGAGTGATGTGTGGTC-3', R: 5'-CTTGTTGTTCACTAGGATGCCG-3'), Nfkbia (F: 5'-GAAGCCGCTGACCATGGAA-3', R: 5'-GATCACAGCCAAGTGGAGTGGA-3'), CD74 (F: 5'-CATGGATGACCAACGCGAC-3', R:5'-TGTACAGAGCTCCACGGCTG-3'), Klf4 (F:

5’-TGCCACTGGTGACCGGATAT-3’, R: 5’-GTCGTTGAACTCCTCGGTCT-3’), Tlr1 (F: 5’-TCAAGTGTGCAGCTGATTGC-3’, R: 5’- TAGTGCTGACGGACACATCC-3’), Irf5 (F: 5’-CCTCAGCCGTACAAGATCTACGA-3’, R: 5’-GTAGCATTCTCTGGAGCTCTTCCT-3’), Irf8 (F: 5’-AGATGGGCCACAGAACGTTA-3’, R: 5’-CCACAAAACTGTCCCATGCA-3’), Il6ra (F: 5’-AGACCTGGGACCCGAGTTAC-3’, R: 5’-AAGGTCAAGCTCCTCCTTCC-3’)，β-actin (F: 5’-CGCCGCTAGAGGTGAAATTCT-3’, R: 5’-CGAACCTCCGACTTTCGTTCT-3’). The following primers are specific for human genes: NFKBIA (F: 5’-CTCCGAGACTTTCGAGGAAATAC-3’, R: 5’-GCCATTGTAGTTGGTAGCCTTCA-3’), CD74 (F: 5’-GACGAGAACGGCAACTATCTG-3’, R: 5’- GTTGGGGAAGACACACCAGC-3’), TLR1 (F: 5’-CCACGTTCCTAAAGACCTATCCC-3’, R: 5’-CCAAGTGCTTGAGGTTCACAG-3’), KLF4 (F: 5’- CCCACATGAAGCGACTTCCC-3’, R: 5’- CAGGTCCAGGAGATCGTTGAA-3’), GATA2 (F: 5’-GCAACCCCTACTATGCCACC-3’, R: 5’-CAGTGGCGTCTTGGAGAAG-3’)，β-ACTIN (F: 5’-TGACATTAAGGAGAAGCTGTGCTAC-3’, R: 5’-GAGTTGAAGGTAGTTTCGTGGATG-3’). The cycling condition is: 95 °C 3 min, (95 °C 15 sec, 60 °C 1 min) x 40 cycles.

Electroporation of human KY821 cells

KY821 cells were passaged 24 hours before electroporation. 1 μg of purified MSCV-GFP or MSCV-GATA2-GFP DNA was electroporated into 2×10^5 cells/20 μl using the Amaxa SE Cell Line Kit (Cat# V4XC-1032, Lonza) and the program CM-137 on a 4D-

Nucleofector (Lonza). After electroporation, cells were plated at 1 X10^6 cells/ml for 24 hours and GFP^+ cells were quantified using hemocytometer and flow cytometry at different time points.

Quantification of nuclear NFkB in human AML cell lines

KY821 and K562 cells were treated with 5 nM bortezomib for 8 hours. During the last 30 minutes treatment, cells were stimulated with 3ng/ml hTNFα (PeproTech, Cat#300-01A). Cells were then spun on a slide and fixed with 4% paraformaldehyde (Invitrogen). Afterwards, cells were blocked with 10% BSA for 10 minutes and incubated with primary antibody against p65 (Cell Signaling Technology, clone D14E12, 1:200) overnight at 4°C. Then, cells were incubated with Alexa 488-conjugated goat anti-rabbit IgG (Invitrogen, Cat# 35552, 1:500). DAPI was used to counterstain the nuclei. The images were analyzed by ImageJ program. The nuclear fraction of p65 in single cell was measured as the intensity from regions overlapped with DAPI staining, and the cytoplasm was calculated by the subtraction of the nuclear intensity from total p65 intensity. The measurement was performed with 250 cells in each group.

Immunohistochemistry (IHC) staining of NFkB p65 in paraffin embedded human BM core samples

Automated immunohistochemistry of NFkB p65 on paraffin-embedded human control and AML BM core slides was performed on the Ventana Discovery Ultra BioMarker Platform (Ventena Medical Systems) at the UWCCC TRIP Lab. Deparaffinization was carried out on the instrument, as well as heat-induced epitope retrieval with citrate-based

cell conditioner 2 buffer (Ventana #950-223) for 56 minutes at 95 ℃. The primary antibody against human NFkB/p65 (Santa Cruz Biotech, Cat# SC-8008) was diluted 1:25 in casein diluent (Ventana, Cat# 760-219) and incubated with slides for 36 min at 37 ℃. Slides were rinsed with reaction buffer (Ventana, Cat# 950-300), incubated with Discovery OmniMap anti-Mouse HRP (Ventana, Cat# 760-4310) for 16 min at 37℃, and then rinsed with a reaction buffer. Discovery ChromoMap DAB Detection Kit (Ventana, Cat#760-159) was used for visualization. Slides were removed from the instrument, counterstained with Harris hematoxylin (1:5) for 45 seconds, rinsed with dH2O, dehydrated by oven drying and dipping in xylene before adding one drop of Mounting medium (Thermo Scientific, Cat#4112) and coverslips were applied to the slides. Total and nuclear p65 levels were quantified using the InForm software (PerkinElmer) in 8,000-18,000 cells per sample.

Supplemental References

1. Zhang J, Wang J, Liu Y, et al. Oncogenic Kras-induced leukemogeneis: hematopoietic stem cells as the initial target and lineage-specific progenitors as the potential targets for final leukemic transformation. *Blood*. 2009;113(6):1304-1314.
2. Wang J, Kong G, Liu Y, et al. Nras G12D/+ promotes leukemogenesis by aberrantly regulating haematopoietic stem cell functions. *Blood*. 2013;121(26):5203-5207.
3. Pietras EM, Reynaud D, Kang YA, et al. Functionally Distinct Subsets of Lineage-Biased Multipotent Progenitors Control Blood Production in Normal and Regenerative Conditions. *Cell Stem Cell*. 2015;17(1):35-46.
4. Wang JY, Liu YG, Li ZY, et al. Endogenous oncogenic Nras mutation promotes aberrant GM-CSF signaling in granulocytic/monocytic precursors in a murine model of chronic myelomonocytic leukemia. *Blood*. 2010;116(26):5991-6002.
5. You X, Liu F, Binder M, et al. Asxl1 loss cooperates with oncogenic Nras in mice to reprogram the immune microenvironment and drive leukemic transformation. *Blood*. 2022;139(7):1066-1079.
6. Zhang J, Kong G, Rajagopalan A, et al. p53-/- synergizes with enhanced NrasG12D signaling to transform megakaryocyte-erythroid progenitors in acute myeloid leukemia. *Blood*. 2017;129(3):358-370.

Supplemental Figure Legends

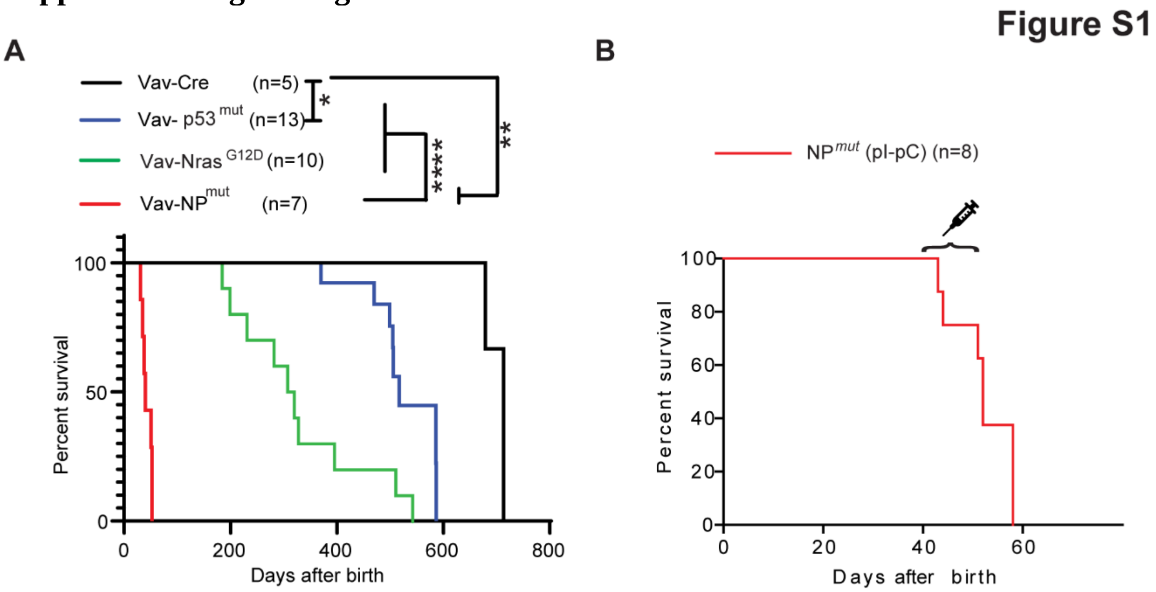

Figure S1. NP^{mut} mice rapidly died when using Vav-Cre (A) or pI-pC injection (B) to activate mutant p53 and oncogenic NRAS. The *Nras* $^{LSL\text{-}G12D/+}$; *Vav-Cre* cohort was previously published [5]. Log-rank test followed by Benjamini-Hochberg multiple comparison analysis was performed. * P<0.05; ** P<0.01; **** P<0.0001.

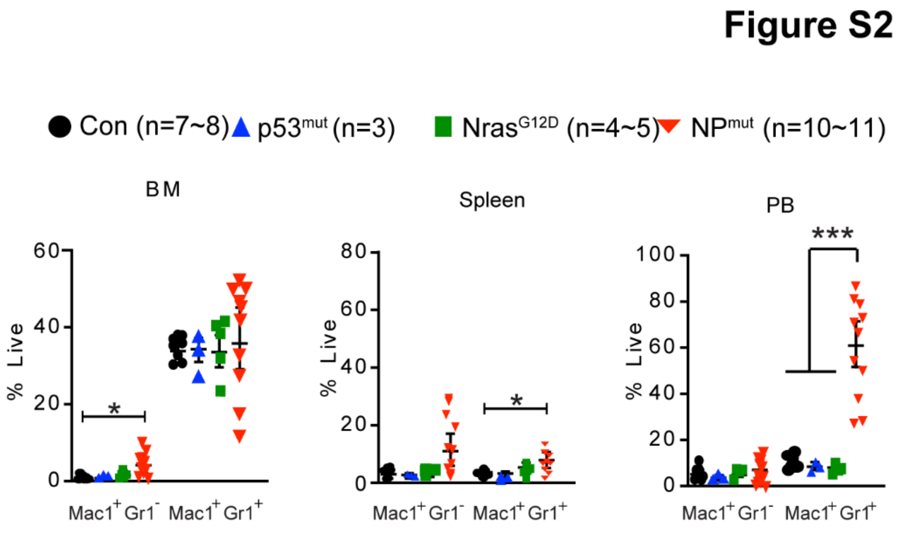

Figure S2. Evaluation of myeloid compartment in moribund NP^{mut} mice and age-matched control, Nras^{G12D}, and p53^{mut} mice. Results are shown as mean ± sd. One-way ANOVA followed by Tukey's post hoc test was performed. * P<0.05; *** P<0.001.

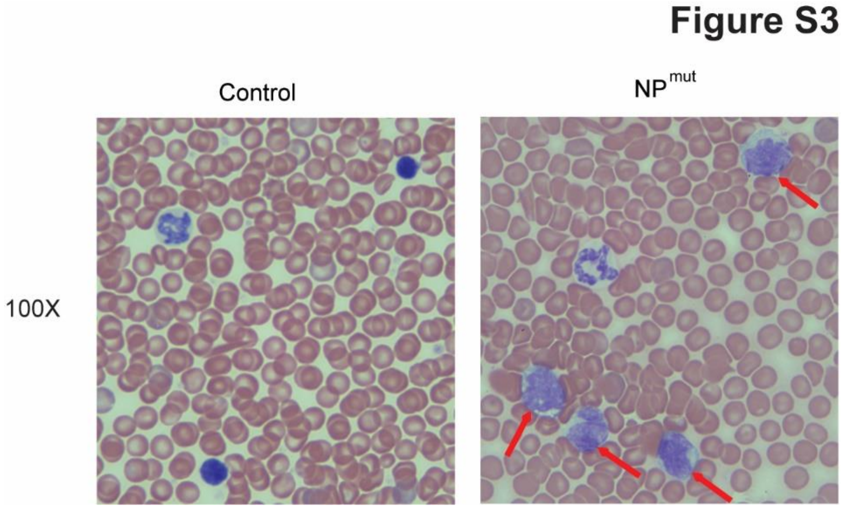

Figure S3. Giemsa staining of blood smears from moribund NP^{mut} mice and age-matched control mice. Arrows indicate atypical, immature monocytoid cells.

Figure S4

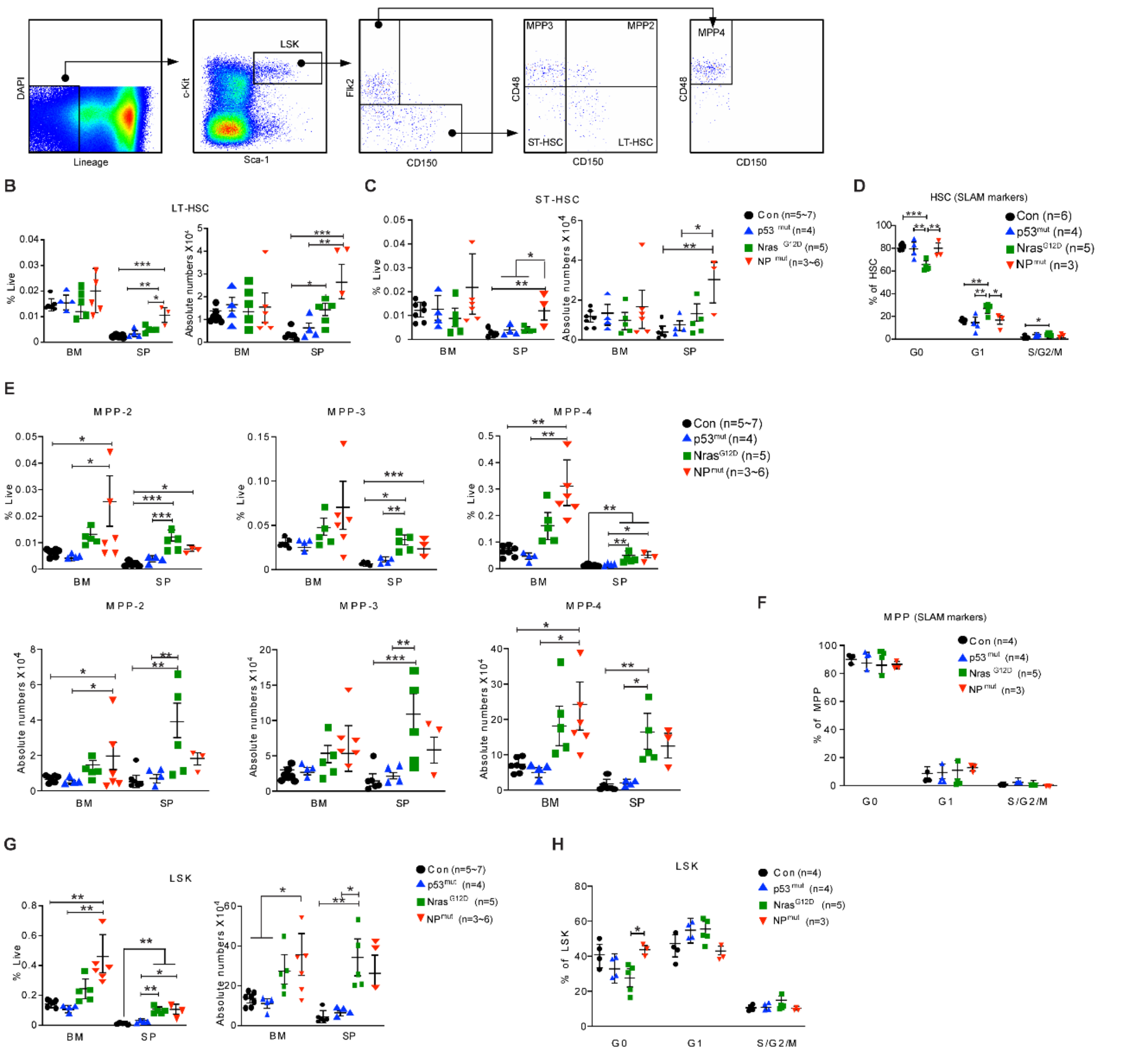

Figure S4. Evaluation of primitive HSPC compartments. Analyses were performed in control, p53^{mut}, and Nras^{G12D} mice 1-week after last pI-pC injection and age-matched NP^{mut} mice. (A) Gating strategy for different populations of primitive HSPCs using control BM cells as an example: Lin$^-$ Sca1$^+$ cKit$^+$ (LSK), long-term hematopoietic stem cell (LT-HSC, defined as Lin$^-$ Sca1$^+$ cKit$^+$ Flk2$^-$ CD48$^-$ CD150$^+$), short-term HSC (ST-HSC, defined as Lin$^-$ Sca1$^+$ cKit$^+$ Flk2$^-$ CD48$^-$ CD150$^-$), multipotent progenitor 2 (MMP2, defined as Lin$^-$ Sca1$^+$ cKit$^+$ Flk2$^-$ CD48$^+$ CD150$^+$), MMP3 (defined as Lin$^-$ Sca1$^+$ cKit$^+$ Flk2$^-$ CD48$^+$ CD150$^-$), and MMP4 (defined as Lin$^-$ Sca1$^+$ cKit$^+$ Flk2$^+$ CD48$^+$ CD150$^-$). (B, C)

Quantification of LT-HSCs (B) and ST-HSCs (C) in bone marrow (BM) and spleen (SP). (D) Cell cycle analysis of total BM HSCs (defined as Lin^- $Sca1^+$ $cKit^+$ $CD48^-$ $CD150^+$). (E) Quantification of MPP2-4 in BM and SP. (F) Cell cycle analysis of total BM MPPs (defined as Lin^- $Sca1^+$ $cKit^+$ $CD48^-$ $CD150^-$). (G) Quantification of LSK cells in BM and SP. (H) Cell cycle analysis of BM LSK cells. (B-H) Results are shown as mean ± sd. One-way ANOVA followed by Tukey's post-hoc test was performed. * $P<0.05$; ** $P<0.01$; *** $P<0.001$.

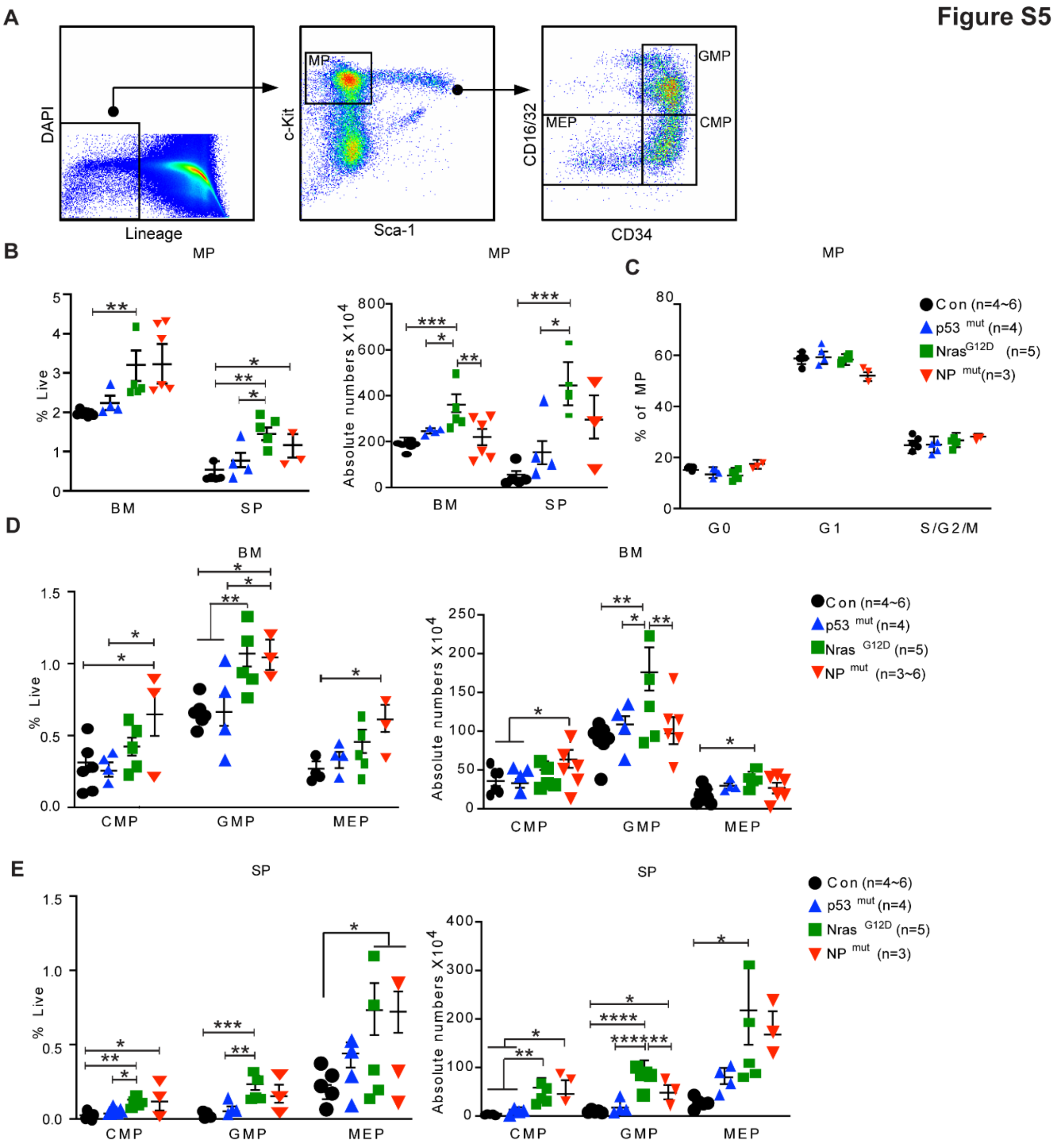

Figure S5. Evaluation of myeloid progenitors (MPs). Analyses were performed in control, p53^{mut}, and Nras^{G12D} mice 1-week after last pI-pC injection and age-matched NP^{mut} mice. (A) Gating strategy for different populations of myeloid progenitors (MPs, defined as Lin$^-$ Sca1$^-$ cKit$^+$): common myeloid progenitor (CMP, defined as Lin$^-$ Sca1$^-$ cKit$^+$ CD34$^+$ CD16/32$^-$), megakaryocyte erythroid progenitor (MEP, defined as Lin$^-$ Sca1$^-$ cKit$^+$ CD34$^-$ CD16/32$^-$), and granulocyte macrophage progenitor (GMP, defined as Lin$^-$ Sca1$^-$ cKit$^+$

CD34+ CD16/32+). (B) Quantification of total MPs in bone marrow (BM) and spleen (SP). (C) Cell cycle analysis of total BM MPs. (D, E) Quantification of CMP, MEP, and GMP in BM (D) and SP (E) as previously described [6]. (B-E) Results are shown as mean ± sd. One-way ANOVA followed by Tukey's post hoc test was performed. * P<0.05; ** P<0.01; *** P<0.001; **** P<0.0001.

Figure S6

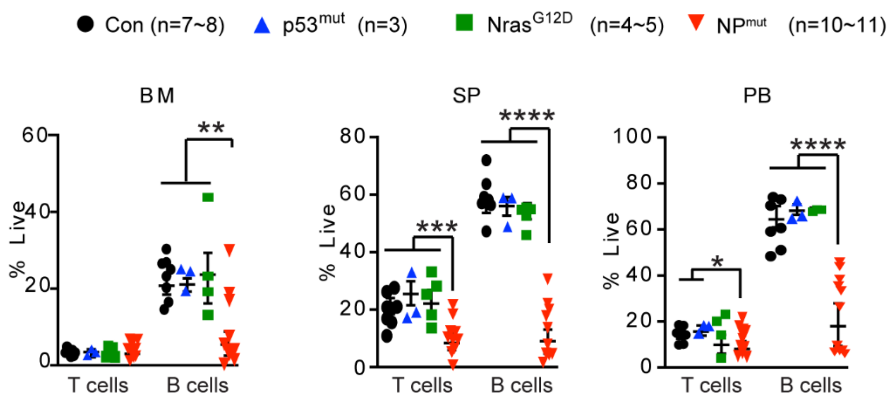

Figure S6. Reduced lymphopoiesis in NPmut mice. Analyses were performed in moribund NPmut mice and age-matched control (Con), NrasG12D, and p53mut mice. Quantification of T- and B-cells in bone marrow (BM), spleen (SP), and peripheral blood (PB). Results are shown as mean ± sd. One-way ANOVA followed by Tukey's post hoc test was performed. * P<0.05; *** P<0.001.

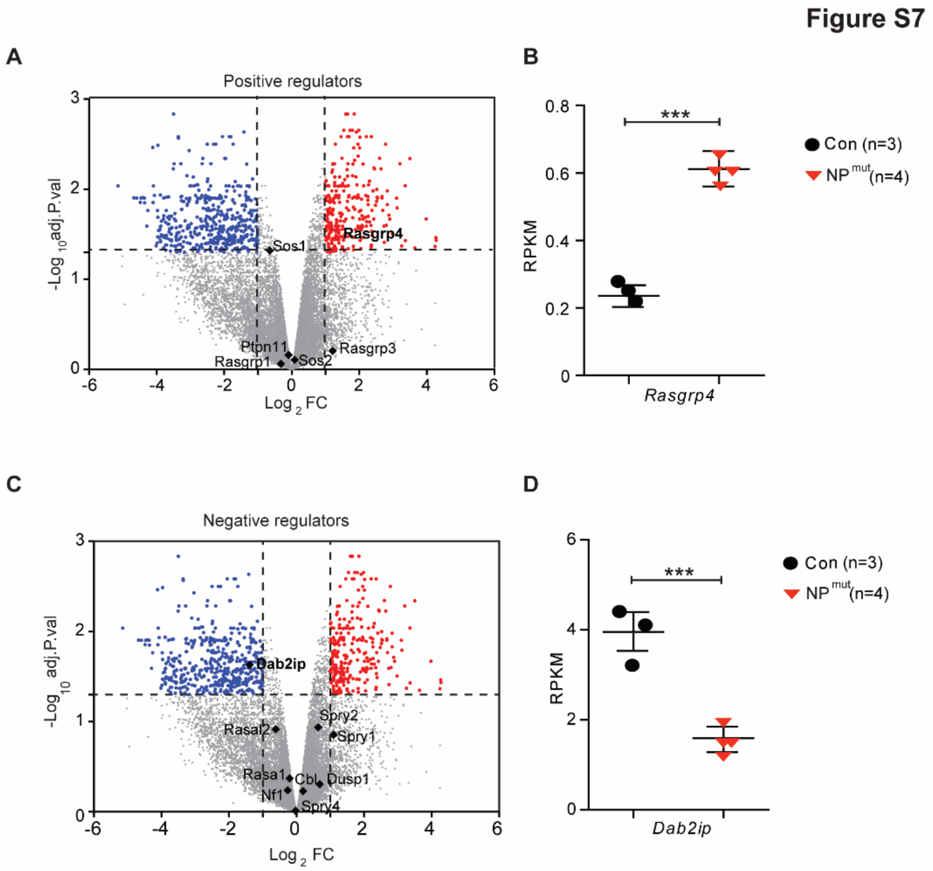

Figure S7. Transcriptional levels of positive and negative regulators of RAS signaling pathway. (A) Volcano plot of positive regulators in NP^{mut} vs control HSPCs (upregulated genes in red and down-regulated genes in blue). (B) Quantification of *Rasgrp4*

transcriptional level. RPKM, reads per kilobase per million mapped reads. (C) Volcano plot of negative regulators in NP^{mut} vs control HSPCs (upregulated genes in red and down-regulated genes in blue). (D) Quantification of *Dab2ip* transcriptional level. (B, D) Results are shown as mean $\pm$ sd. Wald tests within DESeq2 were conducted to assess differential gene expression between groups. P values from differential gene expression analyses were corrected for multiple testing using the Benjamini-Hochberg method. *** P<0.001.

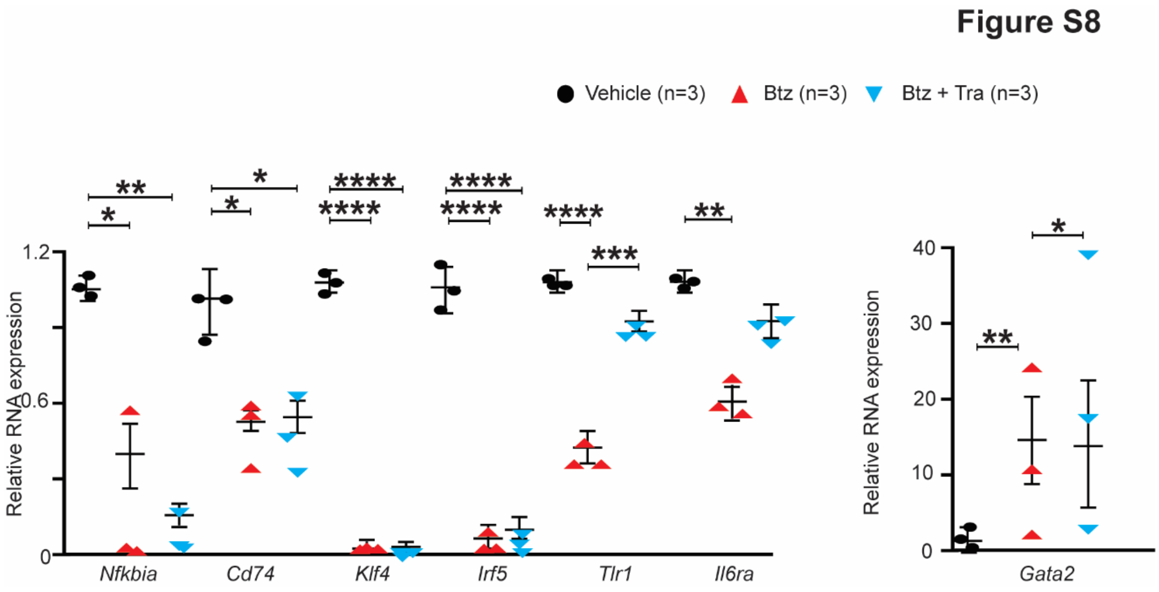

Figure S8. Btz and combo treatment downregulate inflammation gene expression in NP^{mut} leukemia cells. NP^{mut} cells were transplanted into sublethally irradiated CD45.1^{+} recipients. Once AML was established, the recipients were treated with vehicle, Btz, or combined Tra and Btz until the vehicle treated mice became moribund. Donor-derived leukemia cells were flow sorted. The expression levels of inflammation-related genes were quantified using qRT-PCR. Results are shown as mean $\pm$ sd. One-way ANOVA followed by Tukey's post-hoc test was performed. * P<0.05; ** P<0.01; *** P<0.001; **** P<0.0001.

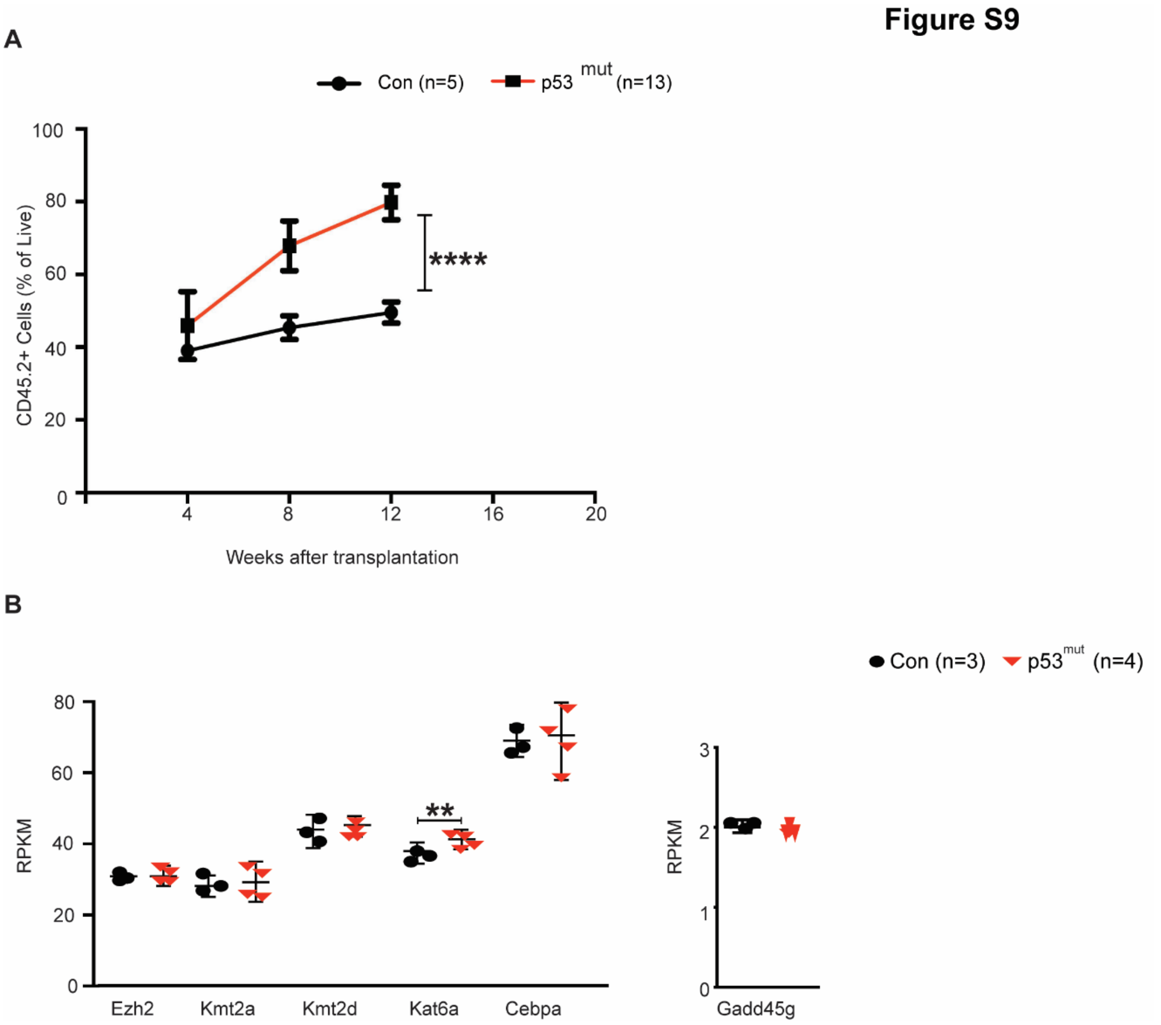

Figure S9. p53^{R172H} confers increased BM reconstitution through mechanisms distinct from p53^{R248W}. (A) Total BM cells were isolated from control and p53^{mut} mice 1-week after last pI-pC injection and transplanted with the same number of competitor cells into irradiated recipients. Donor-derived cells were monitored in the peripheral blood of recipient mice. (B) Quantification of expression levels of several genes in p53^{R172H} vs control cells. These genes are known to be differentially expressed in p53^{R248W} HSPCs. (A, B) Results are shown as mean $\pm$ sd. (A) Two-way ANOVA followed by Bonferroni's multiple comparison test was performed. (B) Wald tests within DESeq2 were conducted to assess differential gene expression between groups. P values from differential gene

expression analyses were corrected for multiple testing using the Benjamini-Hochberg method. ** P<0.01; **** P<0.0001.

Table S2. Evaluation of NP^{mut} HSPCs in initiating AML in recipient mice

Donor cell type	No. of donor cells	No. of helper cells	No. of recipient mice	% Diseased animals
WBM (moribund)	0.25 M	0.25 M	9	100
WBM (6 wk)	0.25 M	0.25 M	5	100
LSK (6 wk)	4000	0.25 M	4	100
MPP2 (6 wk)	50	0.25 M	4	100
MPP3 (6 wk)	100	0.25 M	4	100
MPP4 (6 wk)	700	0.25 M	5	100
GMP (6 wk)	4000	0.25 M	4	50
MEP (6 wk)	2000	0.25 M	5	60
